# A robust phylogenomic framework supports a revised intrafamilial classification of Urticaceae

**DOI:** 10.1016/j.pld.2025.12.003

**Published:** 2025-12-17

**Authors:** Xiao-Gang Fu, Jie Liu, Richard I. Milne, Alex K. Monro, Shui-Yin Liu, Qin Tian, Gregory W. Stull, Amos Kipkoech, Ting-Shuang Yi, De-Zhu Li, Zeng-Yuan Wu

**Affiliations:** a Germplasm Bank of Wild Species & Yunnan Key Laboratory of Crop Wild Relatives Omics, Kunming Institute of Botany, Chinese Academy of Sciences, Kunming 650201, China; b University of Chinese Academy of Sciences, Beijing 100049, China; c Key Laboratory for Plant and Biodiversity of East Asia, Kunming Institute of Botany, Chinese Academy of Sciences, Kunming 650201, China; d Institute of Molecular Plant Sciences, School of Biological Sciences, University of Edinburgh, Edinburgh EH9 3JH, UK; e Identification & Naming Department, Royal Botanic Gardens, Kew, Richmond, Surrey, TW9 3AE, UK; f Center for Interdisciplinary Biodiversity Research & College of Forestry, Shandong Agricultural University, Tai’an 271018, China; g Department of Botany, National Museum of Natural History, Smithsonian Institution, Washington, DC 20013, USA

**Keywords:** *Chiajuia*, Intrafamilial classification, Phylogenomics, Plastome, Sarcochlamydeae, Urticaceae

## Abstract

Over the past decade, phylogenomics has significantly enhanced our understanding of relationships among numerous angiosperm lineages. However, comprehensive phylogenetic studies combining broad sampling of both genomic sequences and taxa within the nettle family (Urticaceae) are still lacking. Here, we reconstructed the phylogeny of Urticaceae (345 species across 89% of accepted genera) using concatenated and coalescent analyses from plastome and nuclear ribosomal DNA sequences. Different plastid datasets and tree inference methods yielded a consistent phylogenetic backbone, with 98% of nodes achieving > 90% bootstrap support — a significant improvement compared to 54% of nodes in the latest published phylogenetic study of Urticaceae. Plastid and nuclear phylogenetic relationships were largely congruent, with several exceptions that warrant further study. In the context of the updated phylogenetic relationships, we propose dividing the family into seven tribes that correspond to seven major clades or subclades, including a newly established tribe, Sarcochlamydeae *stat. nov*. Our phylogenetic analysis indicates that *Debregeasia* and *Phenax* are non-monophyletic. By combing morphological, molecular and distributional evidence, we describe a new genus *Chiajuia gen*. *nov*. Additionally, we propose synonymizing the following genera: *Cypholophus* (to *Boehmeria*), *Haroldiella* (to *Pilea*), *Hemistylus*, *Neodistemon*, *Rousselia* (all to *Pouzolzia*), *Hesperocnide* (to *Urtica*), and *Pellionia* (to *Elatostema*), while recognizing *Elatostematoides*, *Gonostegia*, *Leptocnide*, *Margarocarpus*, *Scepocarpus*, and *Sceptrocnide* as distinct genera. This robust phylogenomic framework and revised classification lays a foundation for future studies on the evolution and ecology of Urticaceae. The approach applied here may also serve as an important reference for other large plant families in angiosperms.

## Highlights

• The most comprehensive phylogenetic framework of Urticaceae.
• Relationships among genera were clarified; and non-monophyletic genera have been well defined.
• The seventh tribe, Sarcochlamydeae, was erected in Urticaceae; and a new genus *Chiajuia* was described.

## 1 Introduction

A robust phylogenetic framework is an essential basis for understanding evolutionary relationships and the diversification of species (Soltis and Soltis, 2000; Delsuc et al., 2005; Pyron, 2015; Smith et al., 2020). Over the past two decades, rapid advancements in next-generation sequencing have generated unprecedented amounts of DNA data. This wealth of information has been valuable in resolving relationships across various evolutionary scales, elucidating spatiotemporal evolutionary patterns and the drivers of diversification within numerous plant groups (Guo et al., 2023). The plastid genome (plastome) is an ideal source of molecular data for phylogenetic studies (Martin et al., 2005; Li et al., 2021). Plastomes are inherited uniparentally, reducing recombination, have a conserved quadripartite structure and protein-coding genes, and are relatively small, with high copy numbers, making them easier to sequence (Staats et al., 2013; Twyford and Ness, 2017). Plastid phylogenomics has effectively resolved intractable intrafamilial relationships in families such as Rosaceae (Zhang et al., 2017b), Fabaceae (Zhang et al., 2020), Lamiaceae (Zhao et al., 2021), and Arecaceae (Yao et al., 2023). Nevertheless, many large families still have ambiguous intrafamilial phylogenetic relationships, especially those that have experienced rapid radiation, which complicates intrafamilial classification.

Urticaceae, first described by de Jussieu (1789), comprises 54 genera and 2625 species, making it one of the largest families of angiosperm (Stevens, 2017). The natural distribution of Urticaceae is subcosmopolitan, with a center of diversity in tropical regions, particularly in tropical Asia, and much lower diversity in temperate zones (Friis, 1989). Morphologically, Urticaceae exhibit considerable diversity: their habits range from tiny herbs and lianas to shrubs or trees; their inflorescences may be cymose, paniculate, racemose, spicate, or cluster-capitate in form; and their flowers are minute, complex, and highly diverse in stigma form (Chen, 1985; Friis, 1989, 1993b; Chen et al., 2003). The family is economically important for fiber production (Lanzilao et al., 2016; Subedee et al., 2020; Wu et al., 2024) and holds potential for pharmacological and medicinal applications (Doukkali et al., 2015; Dhouibi et al., 2020).

**Fig. 1:**
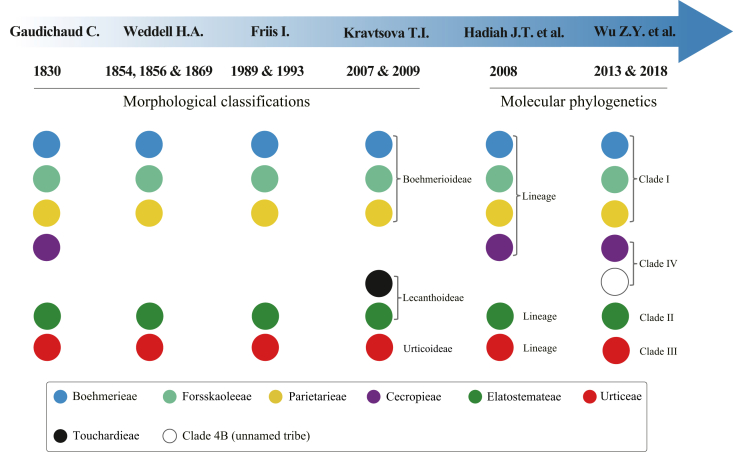
Summary of the main classification schemes for Urticaceae from morphological classifications to present molecular studies. Above the tribal level, Kravtsova (2007, 2009) recognized subfamilies, Hadiah et al. (2008) used three lineages to represent the major clades, and Wu et al. (2013, 2018) designated Clades I–IV to represent the four major clades within Urticaceae. The genera included in each tribe or clade under the respective systems are listed in Table S1.

Intrafamilial classifications of Urticaceae were initially conducted based on morphological characters, such as patterns of ovules and embryos (Gaudichaud, 1830), cystolith morphology (Weddell, 1854, 1856, 1869), carpology (Kravtsova, 2007, 2009), and inflorescence structure (Friis, 1989, 1993b). Six tribes (Boehmerieae, Cecropieae, Elatostemateae, Forsskaoleeae, Parietarieae, and Urticeae) are currently recognized, although circumscriptions vary among different classification schemes (Conn and Hadiah, 2009) (Fig. 1 and Table S1). To date, most molecular phylogenetic studies of Urticaceae have focused on the genus or tribe level, with only three phylogenetic studies conducted at the family level (Hadiah et al., 2008; Wu et al., 2013, 2018). Wu et al. (2018) performed the most comprehensive analysis, utilizing seven loci from 298 individuals representing 258 species across 52 genera. Additionally, a full angiosperm phylogeny based on 353 nuclear genes included 73 species representing 52 genera of Urticaceae (Baker et al., 2022). Monro et al. (2025) combined these data with nine other loci for 532 Urticaceae accessions, proposing two new tribes (Leukosykeae and Myriocarpeae) and two new genera (*Muimar* and *Pouzolziella*), and confirming previous results (Wu et al., 2013, 2018) that resolved four main clades within the family. Despite these efforts, a robust phylogenetic framework for this family is still lacking, which is crucial for resolving its taxonomic issues.

The circumscription and placement of the tribe Cecropieae was for a long time controversial, due to its possessing certain intermediate morphologies between Urticaceae and Moraceae (Fig. 1). Berg (1978) elevated it to family level as Cecropiaceae, based on its straight stamens in bud and its arborescent habit. However, molecular phylogenetic studies have consistently confirmed that the tribe Cecropieae belongs to Urticaceae, and comprises five genera (i.e., *Cecropia*, *Coussapoa*, *Musanga*, *Myrianthus*, and *Pourouma*) (Treiber et al., 2016; Wu et al., 2018; Monro et al., 2025). However, possibly due to the use of limited molecular markers, none of them have resolved the intergeneric relationships within this tribe, even though they examined all five genera. Furthermore, *Debregeasia wallichiana* Wedd., was recently shown to be closed related to the tribe Cecropieae (Kipkoech et al., 2025). Therefore, whether the tribe should be expanded to include this species requires further investigation. Elsewhere in Urticaceae, the phylogenetic position of the monotypic genus *Sarcochlamys* differs substantially among studies: Wu et al. (2018) and Monro et al. (2025) supported its inclusion within Boehmerieae (close to *Boehmeria* and *Archiboehmeria*); whereas Baker et al. (2022) placed it in an isolated clade (together with three other genera: *Gibbsia*, *Leucosyke*, and *Maoutia*, which are currently not assigned to any recognized tribe). Moreover, the delimitation of genera previously determined to be non-monophyletic (Wu et al., 2018), such as *Boehmeria*, *Laportea*, *Pellionia*, *Pouzolzia*, and *Urera*, requires further investigation with wider sampling of both taxa and genomic data. Such efforts will also help determine whether the genera currently regarded as monophyletic are indeed monophyletic, and hence evaluate the validity of the most recent classification revisions within Urticaceae.

In this study, we reconstruct a robust phylogenetic framework for Urticaceae by utilizing plastome and nuclear data based on comprehensive genus-level sampling. We then examine these results together with morphological traits and geographical distributions. Specifically, our objectives were to (1) assess the effectiveness of plastome and nrDNA (18S-ITS1-5.8S-ITS2-26S) data in resolving intertribal and intergeneric phylogenetic relationships within Urticaceae; (2) provide new insights into relationships and circumscriptions of the tribes and genera of the family; (3) propose a framework of intrafamilial classification within Urticaceae based on molecular phylogenetic results and morphological evidence.

## 2 Materials and methods

### 2.1 Taxon sampling and DNA sequencing

We sampled 485 individuals, representing 345 species from 54 genera in Urticaceae. These taxa span across 48 accepted genera, three synonymized genera (*Gonostegia*, *Hesperocnide*, and *Pellionia*), and one taxonomic status unresolved name (*Metatrophis*) following the classification of Stevens (2017). We also included two genera *Haroldiella* and *Urticastrum* that were not recognized by Stevens (2017). Of the individuals examined, 303 individuals (228 species and 39 genera) were newly sequenced (see Table S2, q.v. for voucher specimens and their locations), and 21 accessions (17 species) were added from raw data downloaded via Kew Tree of Life Explorer (PAFTOL project; https://treeoflife.kew.org/). We also incorporated 161 complete or nearly complete plastid genomes, representing 100 additional species, from our previous work (Wang et al., 2020; Ogoma et al., 2022; Wu et al., 2022) and NCBI (last accessed on April 18, 2023; https://www.ncbi.nlm.nih.gov). For outgroups, we downloaded plastomes of 36 species from NCBI, representing four families within Rosales (Moraceae, Cannabaceae, Ulmaceae and Rosaceae) based on Zhang et al. (2011) and Li et al. (2021). The generic names were taken from Stevens (2017) and species nomenclature followed the *Flora of China* (Chen et al., 2003), The Plant List (http://www.theplantlist.org), and recently published literature (Monro et al., 2012; Wilmot-Dear and Friis, 2012; Deng et al., 2013; Wang, 2016; Wang and Wu, 2016; Fu et al., 2021, 2022b) (Table S2).

Genomic DNA was extracted from silica gel-dried leaves or herbarium material using a modified CTAB method (Doyle and Doyle, 1987). DNA samples were then assessed for quality and quantity using a Nano Drop® ND-2000 spectrophotometer (Thermo Fisher Scientific, Wilmington, DE, USA) and 1% agarose gel electrophoresis. For each sample, genomic DNA was fragmented for the construction of a 350 bp short insert library. Library preparation was conducted with NEBNext® Ultra™ II DNA Library Prep Kit for Illumina (New England BioLabs, Ipswich, MA, USA) following the manufacturer's manual, and was subsequently sequenced for 2 × 150 bp paired-end reads on an Illumina HiSeq X-Ten (Illumina, San Diego, CA, USA) at the Laboratory of Molecular Biology, Kunming Institute of Botany, Chinese Academy of Sciences. Approximately 1–10 Gb of raw data were expected to be produced for each sample.

### 2.2 Sequence assembly

Raw sequence data were initially filtered using Trimmomatic v.0.36 (Bolger et al., 2014) with default parameters to obtain high-quality clean reads (Phred score = 33; ILLUMINACLIP: TruSeq3-PE.fa:2:30:10 LEADING:3 TRAILING:3 SLIDINGWINDOW:4:15 MINLEN:36). Then, we used GetOrganelle v.1.7.6.1 (Jin et al., 2020) to conduct *de novo* plastome and nrDNA assembly with the settings as “-R 25 -k 21,35,45,65,85,105,115,127 -F embplant_pt” and “-R 25 -k 21,45,65,85,105,127 -F embplant_nr”, respectively. The chloroplast coding DNA sequences (CDS) were extracted from the assembled plastomes (PT) using PhyloHerb v.1.1.1 (Cai et al., 2022), resulting in the CDS-PT dataset. In addition, we used HybPiper v.2.0.1 (Johnson et al., 2016) to assemble chloroplast CDS for 27 samples that failed to be assembled using GetOrganelle. For reference, we used 87 CDS extracted from the plastome of *Debregeasia orientalis* C.J. Chen (GenBank accession number NC_041413). Specifically, we used BWA v.0.7.17 (Li and Durbin, 2009) for reads mapping. The binned reads were assembled separately for each gene using SPAdes v.3.15.3 (Bankevich et al., 2012), and then the assembled contigs were aligned to the reference DNA sequences to obtain the final CDS using Exonerate v.2.4.0 (Slater and Birney, 2005). We utilized the commands “hybpiper retrieve_sequences” and “hybpiper stats” within HybPiper to retrieve all assembled genes and summarize their recovery from each sample, respectively. These genes were combined with the CDS-PT dataset to generate the final CDS dataset. To reduce missing data, we newly assembled eight nrDNA sequences with GetOrganelle from raw data of the same individuals used for the plastome data, downloaded in each case from the Sequence Read Archive database. Additionally, 15 Urticaceae individuals and 24 outgroups that had plastome data were excluded from the final nrDNA dataset because raw genome data were unavailable (although plastome data existed).

### 2.3 Sequence alignment and dataset generation

Alignments of PT, CDS, and nrDNA were performed using MAFFT v.7.487 (Katoh and Standley, 2013) with the parameters set as “--genafpair --maxiterate 1000”. Columns in each alignment with more than 70% missing data were pruned with “pxclsq” function in Phyx v.1.1 (Brown et al., 2017). Outlier sequences from individual samples were identified and removed using Spruceup v.2022.2.4 (Borowiec, 2019) with default parameters (window size = 20 bp, overlap = 15 bp, criterion = lognormal distribution) and a cutoff threshold value of 0.95. As the efficiency of the Spruceup algorithm improves with larger datasets, all CDS from the matrix were concatenated into a single super matrix using the script “concatenate_fasta.py” (https://github.com/Kinggerm/PersonalUtilities) (Zhang et al., 2020) before running Spruceup. The processed super matrix was then split into single CDS alignments using AMAS (Borowiec, 2016). To decrease the potential effect of missing data without reducing the number of gene loci, we filtered sequences shorter than 90 bp or less than 10% of the gene’s average length. Processed alignments were manually inspected in Geneious v.9.0.2 (Kearse et al., 2012), and the summary statistics were calculated using AMAS. Finally, four datasets were constructed: (1) a complete or nearly complete plastome (PT) matrix (including both inverted repeat regions to preserve the full genomic structure and maximize available phylogenetic signal), comprising 458 Urticaceae individuals (50 genera and 323 species) plus 36 outgroups; (2) a CDS matrix of 485 Urticaceae individuals (54 genera and 345 species) plus 36 outgroups; (3) a nrDNA matrix with 470 Urticaceae individuals (54 genera and 337 species) plus 12 outgroups; (4) a concatenated CDS and nrDNA matrix (CDS + nrDNA), comprising 485 Urticaceae individuals (54 genera and 345 species) plus 36 outgroups. This last dataset enabled the inclusion of individuals with partial or missing nrDNA data.

### 2.4 Phylogenetic inference

We employed the maximum likelihood (ML) method to reconstruct phylogenetic trees from all datasets (PT, CDS, nrDNA, and CDS + nrDNA). Using PartitionFinder v.2.1.1 (all models, AICc criterion and the greedy algorithm) (Lanfear et al., 2017) and ModelFinder (-m MFP) (Kalyaanamoorthy et al., 2017), the best-fitting partition scheme in the CDS dataset was determined to be “partitioned by loci”. ML analyses were implemented in RAxML v.8.2.12 (Stamatakis, 2014) with the default parameters and a GTR + G model. For the best-scoring ML tree, search began from random trees, and branch support was assessed using the “-f a” option with 1000 rapid bootstrap (BS) replicates. Given that the length of the nrDNA matrix is much less than that of the CDS matrix, we treated the CDS + nrDNA dataset as a single locus, and directly inferred the ML trees. In addition, as an alternative method to verify our results, we performed ML analyses using IQ-TREE v.1.6.12 (Nguyen et al., 2015) for each dataset. Branch support was estimated using ultrafast bootstrapping (UFBoot) with 1000 replicates (Hoang et al., 2018).

Additionally, many recent studies have reported significant conflicts within the plastome data (e.g., Gonçalves et al., 2019; Walker et al., 2019; Zhang et al., 2020; Yang et al., 2021), emphasizing the need to account for gene tree heterogeneity when inferring species trees. To address this, we performed phylogenetic analysis with the coalescent-based method using ASTRAL-III v.5.6.3 (Zhang et al., 2018) and compared the results with the concatenated method for the CDS dataset. Prior to ASTRAL analyses, an individual gene tree of 87 CDS was inferred by RAxML with the GTR + G model and 100 rapid bootstraps. Poorly supported branches (BS support less than 10%) were then collapsed using the “nw_ed” program in newick utils v.1.6 (Junier and Zdobnov, 2010), which is believed to improve the accuracy of tree inference (Zhang et al., 2017a). Branch support of the inferred ASTRAL tree was estimated using local posterior probabilities (LPP; Sayyari and Mirarab, 2016). All resulting trees were visualized in FigTree v.1.4.3 (http://tree.bio.ed.ac.uk/software/figtree/) and manually edited using Adobe Illustrator 2025. The comparison of different trees was visualized using the “cophylo” function in Phytools v.0.7.80 package (Revell, 2012). We defined a branch with BS > 90%, UFBoot > 95%, and LPP > 0.95 as strongly supported, whereas those with 90% ≥ BS ≥ 70%, 95% ≥ UFBoot ≥ 90%, and 0.95 ≥ LPP ≥ 0.90 were considered as moderately supported, and those with BS < 70%, UFBoot < 90%, and LPP < 0.90 as poorly supported (Soto Gomez et al., 2019; Pardo-De la Hoz et al., 2023).

### 2.5 Conflict analyses

We employed the bipartition method implemented in PhyParts v.0.0.1 (Smith et al., 2015) to quantify concordant and conflicting bipartitions between 87 chloroplast CDS gene trees and species trees derived from the CDS dataset, as estimated by RAxML and ASTRAL. Initially, we rooted each gene tree and the species trees using Rosaceae species as an outgroup via the “pxrr” function in Phyx. We then mapped bipartitions from all gene trees with BS support at least 50%, which was considered to be informative following several previous studies (e.g., Koenen et al., 2020; Hou et al., 2022; Morales-Briones et al., 2022; Nie et al., 2023) against the RAxML and the ASTRAL tree with PhyParts. Output results were visualized using the script “phypartspiecharts_missing_uninformative.py” (https://bitbucket.org/dfmoralesb/target_enrichment_orthology/src/master/).

## 3 Results

### 3.1 Dataset features

The alignment length of the plastome (PT) data matrix is 375,346 bp and the GC content ranged from 35.20% to 38.70% (Table S3). After removing ambiguous sites and outlier sequences, the alignment length of the PT matrix is 157,710 bp, with 51.40% variable sites, 45.80% parsimony informative (PI) sites, and 5.25% missing data (Table 1). Recovery efficiencies and sequence length of each CDS assembled by HybPiper are shown in Table S4 and Fig. S1. The concatenated CDS matrix (after cleaning) has an aligned length of 93,808 bp, with 45.70% variable sites, 40.10% PI sites, 8.23% missing data, and 37.80% GC content (Table 1). The summary statistics for each of these variables for each alignment of CDS, nrDNA and CDS + nrDNA, are provided in Tables S3 and 1.

**Table 1:** A summary of four datasets used in this study.

Alignment_name	No_of_taxa	Alignment_length	Missing_percent	Proportion_variable_sites	Proportion_PI	GC_content
Plastome	494	157,710	5.245	0.514	0.458	0.366
CDS	521	93,808	8.227	0.457	0.401	0.378
nrDNA	482	5864	5.659	0.345	0.288	0.544
CDS + nrDNA	521	99,672	8.492	0.451	0.395	0.388

### 3.2 Phylogenetic relationships within Urticaceae

The monophyly of genera and high-level clades, as well as their relationships, are generally congruent between plastid datasets (Fig. 2a, Fig. 2b, S2 and S3) and the nrDNA dataset (Fig. S4), with exceptions mostly concerning poorly supported nodes within the nrDNA tree (Fig. 3). For each concatenated dataset, the maximum likelihood (ML) trees reconstructed using RAxML and IQ-TREE show very few topological conflicts (Fig. 2a, Fig. 2b and S3–S5). Likewise, results from concatenation are largely congruent with those from the coalescent-based method based on the CDS dataset (Fig. S6).

**Fig. 2a:**
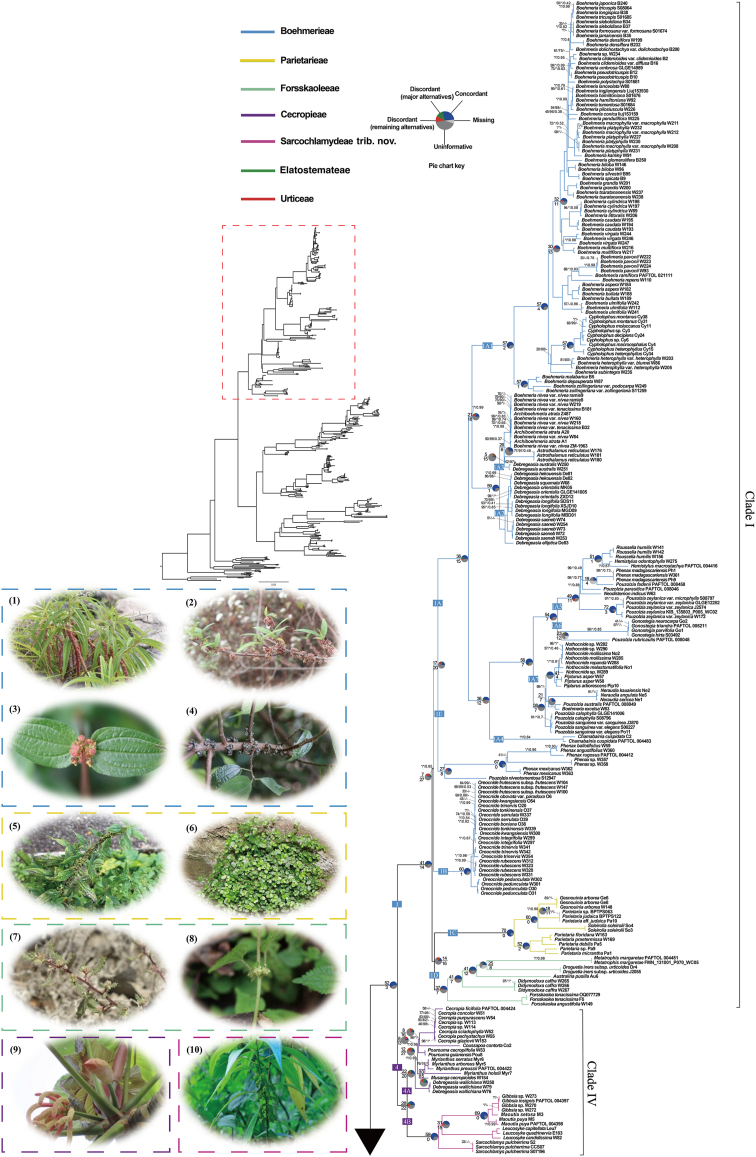
The phylogeny of Urticaceae inferred from maximum likelihood (ML) analysis in RAxML, based on the concatenated 87 chloroplast CDS. Bootstrap support (BS) values (%) from the RAxML analysis, ultrafast bootstrap (UFBoot) values (%) from the IQ-TREE analysis, and local posterior probability (LPP) values from ASTRAL analysis are shown (BS/UFBoot/LPP), whereas a hyphen denotes that this relationship is not recovered by the species tree from the IQ-TREE or ASTRAL analysis. An asterisk indicates the nodes received full support (100%/100%/1.00). Numbers following species name denote the lab codes or data source. Pie charts show whether gene trees are concordant (blue), conflicted (green, a common alternative; red, the remaining alternatives), uninformative (BS < 50%, dark grey), or missing (light grey) for those nodes; numbers above and below the branches indicate the numbers of concordant and conflicting genes at that bipartition, respectively. Representative plant photos were selected for each tribe. (1–4), *Boehmeria penduliflora*, *Debregeasia orientalis*, *Gonostegia hirta*, and *Oreocnide frutescens* in tribe Boehmerieae; (5–6), *Parietaria officinalis* and *Soleirolia soleirolii* in tribe Parietarieae; (7–8), *Forsskaolea angustifolia* and *Droguetia iners* in tribe Forsskaoleeae; (9), *Cecropia peltata* in tribe Cecropieae; (10), *Sarcochlamys pulcherrima* in tribe Sarcochlamydeae *stat*. *nov*.

**Fig. 2b:**
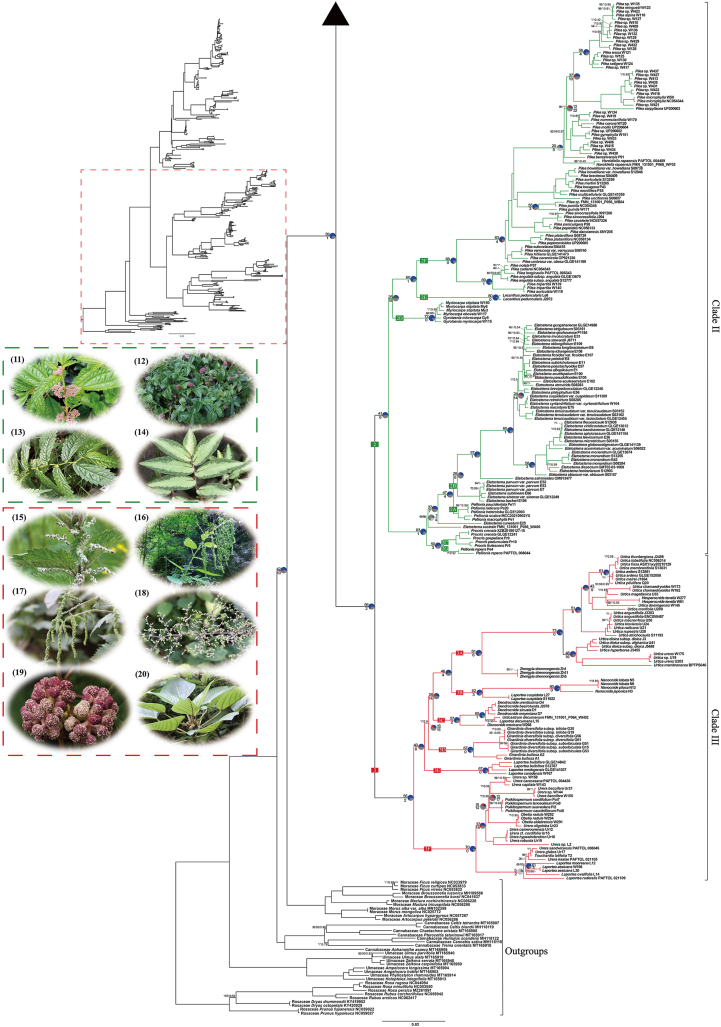
(11–14), *Pilea sinofasciata*, *Lecanthus peduncularis*, *Elatostema umbellatum*, and *Pellionia repens* in tribe Elatostemateae; (15–20), *Urtica dioica*, *Zhengyia shennongensis*, *Girardinia diversifolia*, *Laportea canadensis*, *Poikilospermum suaveolens*, and *Touchardia latifolia* in tribe Urticeae. Photos were taken by Xiao-Gang Fu (1–5, 11–15, 17–18), Zeng-Yuan Wu (6, 16), Jie Liu (7, 9–10), Bing Liu (19), James B. Friday (20), and are used with permission.

**Fig. 3:**
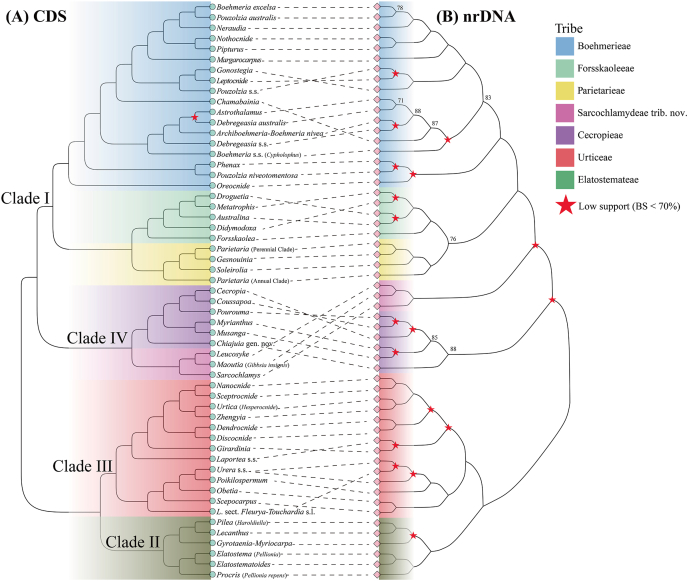
Tanglegram showing topological incongruence between the (A) concatenated 87 chloroplast CDS and (B) nrDNA trees of Urticaceae, both inferred by maximum likelihood (ML) analyses in RAxML. Black dotted lines link corresponding genera or clades between the two topologies. Bootstrap support (BS) values (%) are displayed solely for nodes lacking strong support (BS ≤ 90%). All nodes received strong bootstrap support values (BS > 90%) unless otherwise indicated; nodes receiving low support (BS < 70%) are marked by a red five-pointed star.

The phylogenetic relationships inferred from RAxML based on the concatenated CDS dataset features both the best sampling representation and the highest support values, compared to other trees. These results are therefore used to represent the major phylogenetic findings and are the basis for the following discussion, unless otherwise noted (Fig. 2a, Fig. 2b). The monophyly of Urticaceae, and its division into four main clades (Clades I–IV, with I sister to IV and II sister to III) is fully supported (BS = 100%; UFBoot = 100%; LPP = 1.00) in all trees (Fig. 2a, Fig. 2b), except that Clade IV is supported as non-monophyletic (albeit with low support) in the nrDNA tree (Figs. 3B and S4). All names of clades and subclades follow Wu et al. (2013, 2018) here and thereafter. The main intergeneric relationships within Urticaceae, as inferred from plastid and nrDNA data, are summarized in Fig. 3.

Clade I consists of three tribes (Fig. 2a, Fig. 2b). Of these, Boehmerieae is fully supported as sister to the Forsskaoleeae + Parietarieae clade. Within the tribe Boehmerieae, three subclades (1A, 1B, and 1E) were identified, containing 16 genera in total. Several genera, such as *Boehmeria* (1A1, 1A3, and 1A7), *Debregeasia* (1A2, 1A3, and 4A), *Phenax* (1A5 and 1E), *Pouzolzia* (1A5, 1A6, 1A7, and 1E), and *Archiboehmeria* (1A3), is non-monophyletic. *Oreocnide* (subclade 1B) is sister to the rest of Boehmerieae in the CDS dataset, but the nrDNA trees resolved it as sister to 1E (comprising *Phenax* and *Pouzolzia niveotomentosa* W.T. Wang) with very low support (BS = 46%) (Fig. S4). The tribe Forsskaoleeae (subclade 1D) comprises five monophyletic genera, with *Forsskaolea*, *Didymodoxa*, *Australina*, and *Droguetia* forming successive sister groups to *Metatrophis* (BS = 100%; UFBoot = 100%; LPP = 1.00). Phylogenetic relationships among the latter four genera vary in the nrDNA tree, albeit with mostly low BS support (Fig. S4). Within the tribe Parietarieae (subclade 1C), *Parietaria* is not monophyletic, with two monotypic genera *Gesnouinia* and *Soleirolia* nested within it.

Clade II corresponds precisely to the tribe Elatostemateae, and comprises eight genera. Of these, *Pellionia* is non-monophyletic with species in two different subclades (2A and 2G). *Pilea* (with *Haroldiella*), *Gyrotaenia* (with *Myriocarpa*), and *Elatostema* (with species from subclade 2A of *Pellionia*) are also resolved as non-monophyletic with the genus or clade in parentheses nested within them. Intergeneric relationships within Clade II receives strong support at nearly all nodes and showed high congruence between the CDS and nrDNA trees (Fig. 3).

Clade III matches the tribe Urticeae and is divided into six fully supported subclades (3A–3F). Ten of the 13 genera within Urticeae are fully supported as monophyletic, with the exceptions being *Laportea*, *Urera*, and *Urtica*. The members of *Laportea* are scattered across four subclades (3B, 3C, 3E, and 3F), and those of *Urera* are in subclade 3F, mingling with *Poikilospermum*, *Obetia*, *Touchardia*, and several *Laportea* species. *Urtica* is non-monophyletic because *Hesperocnide* is embedded within it.

Clade IV is divided into two subclades: subclade 4A (comprising tribe Cecropieae and *Debregeasia wallichiana* Wedd.) and subclade 4B, which contains four genera, with *Sarcochlamys, Leucosyke*, and *Maoutia* successively sister to *Gibbsia* (BS = 100%; UFBoot = 100%; LPP = 1.00).

### 3.3 Evaluation of gene tree concordance and conflict

According to PhyParts analyses, 59 out of 60 (59/60) informative plastid gene trees support the monophyly of Urticaceae with bootstrap support (BS) values exceeding 50%, whereas the monophyly of Clades I, II, III and IV are supported by 41/55, 41/47, 55/57, and 28/50 plastid gene trees, respectively (Fig. 2a, Fig. 2b). However, many plastid gene trees showed low bootstrap support (BS < 50%) for almost all nodes. Therefore, the high support observed in the concatenated plastid tree is likely driven by a subset of highly informative genes. Among these four clades within Urticaceae, Clade I shows relatively high levels of conflict among gene trees at certain nodes, such as the stem node of the largest subclade 1A (with 17/37 informative plastid genes), the stem node of *Oreocnide* (1B) (with 7/34 informative plastid genes), and the crown node of subclade 1C + 1D (with 14/30 informative plastid genes). Conversely, intergeneric and higher-level relationships within Clade II, Clade III, and Clade IV are largely concordant across most gene trees (Fig. 2a, Fig. 2b).

Three regions within the phylogeny show significant conflicts, involving strong support for conflicting relationships observed at the generic level among 87 plastid gene trees and species trees (the RAxML tree and the ASTRAL tree). First, the sister relationship between the *Nothocnide* + *Pipturus* clade and a clade comprising *Neraudia* and *Pouzolzia australis* (Endl.) Friis & Wilmot-Dear + *Boehmeria excelsa* Wedd. has 99% support in the RAxML tree; however only 8/23 of locally informative (i.e. informative concerning relevant nodes) gene trees support this relationship (Fig. 2a, Fig. 2b). Second, the phylogenetic position of *Oreocnide* (as sister to the rest of Boehmerieae species) has 100% support in the RAxML tree, but only 7/34 locally informative gene trees support this topology (Fig. 2a, Fig. 2b). Finally, the sister relationship between *Coussapoa* and *Cecropia* has 96% support in the RAxML tree, but only 8/24 locally informative gene trees support this relationship (Fig. 2a, Fig. 2b and S7). An alternative arrangement, with *Coussapoa* as sister to the *Cecropia* + *Pourouma* clade in the ASTRAL tree (LPP = 0.99), is supported by a similar number (8/21) of locally informative gene trees (Fig. S2 and S8).

## 4 Discussion

### 4.1 Insights into the plastid phylogenomic resolutions of Urticaceae

Most large-scale phylogenetic analyses using plastomes exclude non-coding regions because they are difficult to align across evolutionarily complex or highly divergent taxa (see Lamiaceae in Zhao et al., 2021). Furthermore, gene rearrangements and inversions complicate the evaluation of orthologs over deep time scales (see angiosperms in Li et al., 2021). Here, the relationships at genus level and above are essentially identical between RAxML analyses of the plastid datasets that included (full plastome) and excluded non-coding regions (CDS-PT dataset, the RAxML inference of this dataset is identical to that of the CDS dataset), indicating a negligible influence of non-coding regions on the phylogenetic results within Urticaceae (Fig. S9). When combining nrDNA data with the CDS dataset, there are very few topological changes, and neither the phylogenetic resolution nor node support value for intergeneric relationships is significantly improved. While support for certain nodes increased (e.g., the stem node of *Astrothalamus*), it decreased for others (e.g., the stem node of *Coussapoa*, and *Haroldiella*) (Fig. 2a, Fig. 2b and S5). Recently, the coalescent-based method with nuclear data has increasingly been used to infer phylogenetic relationships at the family level, even when only 80–90 nuclear genes are examined (which is comparable to the number of plastid CDS genes), e.g. in Elaeagnaceae (83 single nuclear genes; Gu et al., 2024) and Rhamnaceae (89 single nuclear genes; Tian et al., 2024). However, the use of coalescent-based methods for plastid phylogenetic reconstruction is rare due to the assumption of uniparental inheritance and lack of genetic recombination (Birky, 1995). Greater knowledge of plastid inheritance has allowed the documentation of possible biparental inheritance and of distinct evolutionary histories among plastid genes (Corriveau and Coleman, 1988; Walker et al., 2019). In this study, the concatenation method (RAxML) and the coalescent-based method (ASTRAL) produced highly similar phylogenetic trees (Fig. 2a, Fig. 2b and S6). This similarity suggests that genetic recombination has a negligible effect on the phylogeny of Urticaceae based on plastid data. Some inconsistencies observed between the trees generated by the two methods, particularly regarding intergeneric relationships, are likely due to a limited number of informative sites or the amount of missing data. Such discordance occurred at both shallow and deep nodes within the tree. At some nodes, the number of concordant genes and conflicting genes is roughly equal, for example, the stem nodes of Parietarieae (14/16); however, in some instances, the number of conflicting genes substantially exceed the number of concordant genes, for example, the stem nodes of *Oreocnide* (7/27) (Fig. 2a, Fig. 2b). Similar patterns of conflict among plastid genes were also observed in the angiosperm phylogeny by Walker et al. (2019).

The efficacy of plastome data in resolving relationships varies considerably among angiosperm families. For example, Scrophulariaceae (∼56 genera and 2000 species) is comparable to Urticaceae in generic and species diversity. Villaverde et al. (2023) conducted an ML analysis (using IQ-Tree) of 86 plastid CDS genes from 73 individuals representing 66 species from 48 genera of Scrophulariaceae. At both the genus level and higher, 86% (44/51) of nodes were strongly supported (UFBoot > 95%), compared to 100% in the current study of Urticaceae (Fig. 2a, Fig. 2b). The higher resolution in our findings might reflect differing diversification patterns that influence the degree of phylogenetic resolution within the family. To facilitate direct comparisons between studies, we collapsed multiple samples of certain taxa for each genus into a single point on the tree, as shown in Fig. S10. Compared to the most resolved previous tree within Urticaceae of Wu et al. (2018) (based on four chloroplast genes, two nuclear regions, and one mitochondrial gene from 258 species across 52 genera), the proportion of nodes with BS support > 90% in the RAxML tree has increased from 54% (24/44) to 98% (53/54), and the phylogenetic positions of approximately ten clades or genera have changed. In addition, to eliminate the potential effects of increased taxon sampling, we removed those species not included in Wu et al. (2018), including all representatives of *Australina* and *Coussapoa* because the two studies examined different members of these genera; otherwise, all genera remained well-represented. We then reconducted the analysis. This time, the proportion of strongly supported nodes increased from 54% (24/44) to 95% (41/43), confirming that enhanced plastome coverage is the primary factor contributing to the increased phylogenetic resolution (Fig. S11).

### 4.2 Comparison between plastid and nuclear trees of Urticaceae

**Fig. 4:**
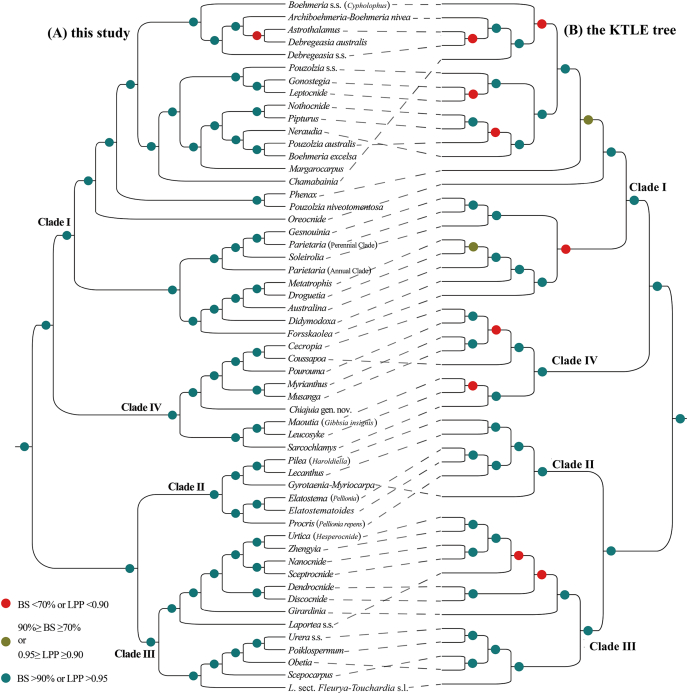
Comparison between the phylogeny inferred from maximum likelihood (ML) analysis in RAxML based on the concatenated 87 chloroplast CDS in this study (A), and the multispecies coalescent tree (the KTLE tree) based on 353 nuclear genes from Baker et al. (2022) (B). Black dotted lines link corresponding genera or clades between the two topologies.

Incongruence between plastid and nuclear trees may indicate reticulate evolution (Rose et al., 2021; Gardner et al., 2023). For Urticaceae, our nuclear phylogenetic tree based on nrDNA is largely congruent with the plastome tree; however, the resolution of intergeneric relationships remains limited. Fortunately, the Kew Tree of Life Explorer (https://treeoflife.kew.org/; Baker et al., 2022), here referred to as KTLE, provided a phylogeny for Urticaceae based on 353 nuclear genes from 74 individuals representing 73 species of 52 genera. The proportion of nodes with BS > 90% and/or LPP > 0.95 is lower (77%, 37/48) in the KTLE tree than our plastid tree (98%, 53/54). Phylogenetic relationships among the same clades or genera are generally concordant between the two trees, with 1–3 discordances per clade, as discussed below (Fig. 4).

Within Clade I, the monotypic genus *Chamabainia* is sister to a clade comprising *Archiboehmeria*, *Boehmeria nivea*, *Astrothalamus*, and *Debregeasia* in both the nrDNA trees and the KTLE tree, which differs significantly from our plastid tree (Fig. 3, Fig. 4). The sole species of *Chamabainia* is herbaceous with opposite leaves, whereas *Boehmeria nivea* and species of *Archiboehmeria*, *Astrothalamus* and *Debregeasia* are mostly shrubs or small trees with alternate leaves (Chen, 1980; Wilmot-Dear, 2009; Wu et al., 2015). Additionally, *Pouzolzia australis* (endemic to Lord Howe, Norfolk and Kermadec Islands) was poorly supported as sister to the *Nothocnide* + *Pipturus* clade (LPP = 0.87), with these together forming a sister clade to *Neraudia* in the KTLE tree (LPP = 1.00). However, both our plastid and nrDNA trees indicate that *P*. *australis* is sister to another geographically isolated species, *Boehmeria excelsa* (endemic to Juan Fernández Islands). Together, they are strongly supported as sister to *Neraudia*, with these together forming a sister clade to the *Nothocnide* + *Pipturus* clade. Because *B. excelsa* was not included in the KTLE tree, we re-ran the RAxML analysis excluding this species, but this produced the same topology as simply pruning *B. excelsa* from our tree. Hence the inclusion of this species did not cause the discordance with the KTLE tree; however wider taxon sampling overall is one possible cause of it.

Within Clade II, the clade comprising *Gyrotaenia* and *Myriocarpa* (subclade 2D) was fully supported as the earliest diverging clade in the KTLE tree. Conversely, our plastid tree, regardless of method, always places this clade as sister to the *Lecanthus* + *Pilea* (with *Haroldiella*) clade, with full support. This is a clear cytonuclear conflict which might reflect ancient hybridization (Wu et al., 2015), and hence requires further investigation.

Within Clade III, the phylogenetic position of *Laportea* species (subclade 3E) is fully resolved in the plastid tree with high support, but it remained unresolved in the KTLE and our nrDNA tree (Fig. 3, Fig. 4B). The phylogenetic relationship between the African genus *Obetia* and the clade comprising all African *Urera* species shows strong cytonuclear discordance. Both nuclear trees (Fig. 3, Fig. 4B) strongly support their sister relationship, whereas the plastid tree (Fig. 2a, Fig. 2b) strongly indicates that the divergence of *Obetia* occurred later. Further research is needed to determine which relationship better represents evolutionary reality.

Within Clade IV, previous studies had confirmed that *Cecropia*, *Coussapoa*, *Musanga*, *Myrianthus*, and *Pourouma* belong to the tribe Cecropieae of Urticaceae, but failed to resolve their intergeneric relationships (Treiber et al., 2016; Wu et al., 2018; Monro et al., 2025). Our work places the three ant-housing, mainly Neotropical genera *Cecropia*, *Coussapoa*, and *Pourouma* (Treiber et al., 2016) in one clade, sister to the antless west African genera *Myrianthus* and *Musanga* (Fig. 3). However, the KTLE recovered a different topology: (((*Cecropia* + *Pourouma*), (*Musanga* + *Myrianthus*)), *Coussapoa*) (Fig. 4B), implying Neotropics to Africa dispersal, whereas our results remain equivocal (the sister species of the clade, *Debregeasia wallichiana*, is from Indo-China).

### 4.3 Principal considerations for a revised tribal level classification

Integrating our phylogenetic results based on plastomic and nrDNA data with previous molecular phylogenetic studies, morphological evidence and geographical distributions, a revised tribal level classification for the Urticaceae is proposed.

#### 4.3.1 Tribe 1. Boehmerieae (Clade I — subclades 1A+1B+1E)

This tribe has long posed taxonomic challenges at the generic and infrageneric levels, especially concerning *Boehmeria* and *Pouzolzia* (Wilmot-Dear and Friis, 1996, 2004, 2013). Here, *Boehmeria* is resolved into three strongly supported monophyletic clades (Fig. 2a, Fig. 2b). The first is *Boehmeria* s.s. (subclade 1A1, comprising most *Boehmeria* species, excluding *B*. *nivea* and *B*. *excelsa*), with *Cypholophus* nested within it. The genus *Cypholophus* comprises about 15 species and is mainly distributed from Malesia to the South Pacific Islands (Friis, 1989, 1993b). Some *Cypholophus* species exhibit morphological features — such as inflorescence architecture, leaf morphology, and geographical distribution — that overlap with those of *Boehmeria* s.s. (Wilmot-Dear and Friis, 1998, 2010; Wilmot-Dear et al., 2010), leading them to sometimes be placed within *Boehmeria*. Weddell (1856) and Friis (1993b) stated that the genus *Cypholophus* can be distinguished from *Boehmeria* by the minute tightly curled style and flesh fruiting perianth. However, the flesh fruiting perianth is hard to observe in specimens (Wilmot-Dear, 2009), and the diagnostic style character is also shared by some *Boehmeria* species, for example, *Boehmeria depauperata* Wedd. and *B*. *zollingeriana* Wedd. (Wilmot-Dear and Friis, 2004). In this study, we sampled nine individuals of *Cypholophus* representing five species, including the type of the generic name, *Cypholophus macrocephalus* Wedd. Our phylogenetic trees based on both plastid and nuclear data consistently indicate that *Cypholophus* forms a monophyletic clade nested within *Boehmeria* s.s., aligning well with the findings of Wu et al. (2018) and Monro et al. (2025). Considering that these two genera share the same filamentous style and fruiting perianths that are hardly detachable from the achenes, we propose synonymizing *Cypholophus* within a re-circumscribed *Boehmeria*. The second clade comprises *Boehmeria excelsa* + *Pouzolzia australis* and is phylogenetically unrelated to either genus. Notably, *P*. *australis* was previously placed in *Boehmeria*, but its shiny fruit, easily detachable fruiting perianth and fruit wings derived from outgrowths of perianth (unlike in *Boehmeria* where the perianth folds to form wings) favors the placement of this species in *Pouzolzia* (Wilmot-Dear and Friis, 2004). However, the densely branched woody habit, the serrate leaves with white tomentose, and the very long persistent style in *P*. *australis* make it quite distinct from any Old or New World species within *Pouzolzia*, whereas these traits are similar to those of *B*. *excelsa* (Wilmot-Dear and Friis, 2004). Hence we support the erection of *Pouzolziella*, proposed by Monro et al. (2025) for *P*. *australis*, but this genus should encompass *B*. *excelsa* as well. The third clade comprises *B*. *nivea* plus *Archiboehmeria atrata*. Our study sampled multiple individuals of both species and confirmed their close relationship: they are indistinguishable based on plastome data and are supported as sisters based on nrDNA data (Fig. S4). Monro et al. (2025) proposed a new genus *Muimar* for *B*. *nivea* but our results suggest that it belongs to *Archiboehmeria*. This issue requires further investigation within the framework of phylogenomics and integrative taxonomy.

Species of *Pouzolzia* are spread across five strongly supported clades (Fig. 2a, Fig. 2b), including the previously mentioned *P*. *australis*. One of these clades can become a single monophyletic clade by merging *Rousselia*, *Hemistylus*, and *Neodistemon* into *Pouzolzia* s.s. (subclade 1A5, defined as species excluding *P*. *australis*, *P*. *niveotomentosa*, *P*. *rubricaulis*, and *P*. *sanguinea*), based on morphology, as discussed by Wu et al. (2013, 2015) and Monro et al. (2025). Additionally, we propose to transfer *Phenax madagascariensis* Leandri into this expanded group based on DNA evidence, as its morphological affinity with *Phenax* has repeatedly been questioned by Friis (1989, 1993a, 1993b). Sister to this expanded *Pouzolzia* s.s. clade is a clade in which *Pouzolzia rubricaulis* (Blume) Wedd. is sister to *Gonostegia*. *Gonostegia* (once included within *Pouzolzia* sect. *Memorialis* in the study of Wilmot-Dear and Friis, 2004) differs from *Pouzolzia* in having opposite leaves with only basal lateral veins. Hence, we concur with Weddell (1869) and Monro et al. (2025) in recommending that *Gonostegia* be maintained as a separate genus, but *P*. *rubricaulis* would be morphologically anomalous within it. However, *P*. *rubricaulis* markedly differs from all other *Pouzolzia* taxa in its very broad caudate stipules with thick texture, tiny stigma, and very small flower but very large fruit (Kravtsova et al., 2003; Wilmot-Dear and Friis, 2004), so we propose to treat it as an independent genus and support the resurrection of the genus *Leptocnide* by Monro et al. (2025). Of the remaining two *Pouzolzia* species examined here, *P*. *niveotomentosa* (endemic to Southwest China) is sister to *Phenax*, but distinct from both genera in its cobwebby tomentose abaxial leaf surfaces, relatively short petioles and unusual spinose female inflorescences (Wilmot-Dear and Friis, 2004), and hence should be assigned to a newly established monotypic genus. Likewise, *Pouzolzia sanguinea* (Blume) Merr. (including the synonymized *P*. *calophylla* W.T. Wang & C.J. Chen) formed an isolated monophyletic clade in the phylogeny. Morphologically, *P. sanguinea* is often a tree with chartaceous or coriaceous bicolored leaves, and vegetatively very variable with a widespread distribution in Asian-Malesian regions. Hence, we support placing this in a separate genus, for which Weddell's (1854) genus *Margarocarpus* might be appropriate (Wilmot-Dear and Friis, 2004; Monro et al., 2025).

The genus *Debregeasia*, recently re-circumscribed to exclude *Debregeasia wallichiana* Wedd. (discussed below) was considered to be monophyletic (Kipkoech et al., 2025), with the newly described Australian species *D. australis* Friis, Wilmot-Dear & C.J. Chen sister to the remaining *Debregeasia* species. However, both plastid and nuclear trees of the current study indicated that *D. australis* is closer to the genus *Astrothalamus,* not sampled by Kipkoech et al. (2025). Therefore, *D*. *australis* may not belong to *Debregeasia*, and further investigation is needed regarding its taxonomic placement, especially with expanded population-level sampling in Australia.

#### 4.3.2 Tribe 2. Parietarieae (Clade I — subclade 1C)

As currently circumscribed within Parietarieae (Friis 1993b; Wu et al., 2015, 2018), this tribe comprises three genera: *Parietaria* is non-monophyletic with respect to *Gesnouinia* and *Soleirolia* (see also Monro et al., 2025). However, if *Parietaria* is divided into two genera, one with annual and the other perennial species, then all four genera become monophyletic as well, at least for species sampled in this study. Both *Gesnouinia* and *Soleirolia* are highly distinctive in habit, and respectively distinct from *Parietaria* in their linear and penicillate stigmas. Considering that the stigma type of female flowers is consistently regarded as a key trait for intergeneric classification within Urticaceae (Chen, 1985; Wu et al., 2015), we support maintaining these as separate genera as previously proposed by Schüßler et al. (2019), rather than merging *Gesnouinia* and *Soleirolia* into *Parietaria* as proposed by Monro et al. (2025). However, only seven species (a few samples have not been identified to the species level) were sampled in the current study, and only three by Monro et al. (2025), out of 20 known species (Friis, 1993b; Wu et al., 2013). Hence, further phylogenetic analysis including more species is needed to test this suggestion.

#### 4.3.3 Tribe 3. Forsskaoleeae (Clade I — subclade 1D)

Within Urticaceae, the tribe Forsskaoleeae is rather distinctive due to its male flowers with only one stamen and a single perianth (Friis, 1989, 1993b). Based on morphology, Friis and Wilmot-Dear (1988) resolved four genera within Forsskaoleeae, i.e. *Australina*, *Didymodoxa*, *Droguetia* and *Forsskaolea*. The genus *Metatrophis* was first described by Brown (1935) and contained only a single species (*Metatrophis margaretae* F. Br.), endemic to Rapa Island, French Polynesia. It was initially placed in Moraceae, but was later transferred to Urticaceae by Florence (1997). Stevens (2017) recognized the genus within Urticaceae; however, its taxonomic status and phylogenetic position remain unclear. In our work, we find *Metatrophis* falls into the tribe Forsskaoleeae (see also Monro et al., 2025). Species of *Metatrophis* share with Forsskaoleeae such traits as inflorescences enclosed by involucre, male flowers boat-shaped with one tepal and one stamen, the lack of pistillode, female flowers with tubular perianth, and a filiform stigma. Hence, morphology and phylogenetic evidence support the inclusion of *Metatrophis* within this tribe.

#### 4.3.4 Tribe 4. Cecropieae (Clade IV — subclade 4A)

Traditionally, the tribe Cecropieae comprises five genera (*Cecropia*, *Coussapoa*, *Musanga*, *Myrianthus*, and *Pourouma*), which are distributed in the Neotropics and Africa. Our results show that *Debregeasia wallichiana* Wedd. (formerly treated within the tribe Boehmerieae) is sister to the Cecropieae clade, in agreement with Kipkoech et al. (2025). Based on our field observations and literature review (Berg, 1978; Wilmot-Dear, 1988; Treiber et al., 2016), we found that *D*. *wallichiana* exhibits many morphological similarities with members of the tribe Cecropieae (for example, *Coussapoa*), such as small tree habit, spirally arranged leaves being crowded towards the apex with very large leaf-scars and stipules, large unisexual inflorescences with long peduncles, tubular and fleshy perianth, and staminate flowers with straight filaments. Accordingly, it is appropriate to consider treating this species as a new genus and transferring it to an expanded Cecropieae tribe. Kipkoech et al. (2025) proposed restoring *D*. *wallichiana* as *Morocarpus wallichiana* (Wedd.) Thwaites. However, upon careful review, we found that *Morocarpus* is a superfluous synonym for *Blitum* (Amaranthaceae) (see https://www.ipni.org/n/329955-2). Therefore, a new genus name for *D*. *wallichiana* is warranted.

#### 4.3.5 Tribe 5. Sarcochlamydeae *trib*. *et stat*. *nov.* (Clade IV — subclade 4B)

We elevate Weddell’s subtribe Sarcochlamydeae (Weddell, 1854) to the tribal level (type: *Sarcochlamys* Gaudich.) to encompass four genera, i.e. *Gibbsia*, *Leucosyke*, *Maoutia* and *Sarcochlamy*, all of which were previously assigned to Boehmerieae (Friis, 1989, 1993b). This contrasts with Monro et al. (2025), who tentatively proposed a new tribal name, Leukosykeae, for this clade. Based on our samples, *Gibbsia insignis* Rendle is nested within *Maoutia*, so *Gibbsia* might be best synonymized into it. However, not all our *Gibbsia* samples could be named to species, and hence the affinities of the only other recognized *Gibbsia* species, *Gibbsia carstenszensis* Rendle (Friis, 1993b), must first be examined before a decision is made. We do not support the proposal to merge both genera into *Leucosyke* (Mabberley, 2017), because despite shared traits such as capitate stigmas, species of *Leucosyke* are distinct in having conspicuous pistillate perianth and a unique receptacle (Friis, 1989; Wilmot-Dear, 2009).

#### 4.3.6 Tribe 6. Elatostemateae (Clade II)

Within Elatostemateae, the phylogenetic position of a clade comprising the Central and South American genera *Gyrotaenia* and *Myriocarpa* has been inconsistent across different phylogenetic studies. Generally, the *Gyrotaenia*-*Myriocarpa* clade either (1) represents the earliest-diverging lineage within the tribe (Wu et al., 2018; Baker et al., 2022; Monro et al., 2025); or (2) forms a sister group to the *Lecanthus* + *Pilea* (including *Haroldiella*) clade, whereas *Elatostema* and its allied genera (*Elatostematoides*, *Pellionia* and *Procris*) are the earliest-diverging lineage (Wu et al., 2013, 2015). The phylogenetic tree based on 353 nuclear genes strongly supported the former topology (Baker et al., 2022), whereas our results based on plastid data fully support the latter (Fig. 2a, Fig. 2b and 3). Monro et al. (2025) placed *Gyrotaenia* and *Myriocarpa* into as a new tribe, Myriocarpeae, which was sister to Elatostemateae. However, under the latter topology, this would render the Elatostemateae non-monophyletic, so we support assigning these two genera to an expanded Elatostemateae. In addition, the presence of linear cystoliths is a significant synapomorphy supporting this placement (Wu et al., 2015). The genus *Haroldiella* was first described by Florence (1997) from French Polynesia, primarily due to its distinctive features relative to *Pilea*, including alternative, spiral phyllotaxy and pinnate leaf venation. However, subsequent research has shown that these characters also occur in other *Pilea* species, such as *Pilea domingensis* Urb. from Hispaniola (Monro, 2006; Jestrow et al., 2012). Recent phylogenetic analyses using several molecular markers support merging *Haroldiella* into *Pilea* (Fu et al., 2022a; Monro et al., 2025), a conclusion further confirmed by our study.

*Elatostema* is one of the most species-rich genera in Urticaceae, with over 500 species (Wang, 2014), but its taxonomic circumscription has long been problematic. Friis (1993b) combined *Pellionia* into *Elatostema* and treated *Procris* as a distinct genus based on morphology. Conn and Hadiah (2011) later transferred *Pellionia repens* (Lour.) Merr. to *Procris*, which is supported by both molecular and morphological phylogenies (Wu et al., 2013, 2015, 2018). Our current data strongly support the monophyly of both *Pellionia* (nested within *Elatostema*) and *Procris* (including *Pellionia repens*) (Fig. 2a, Fig. 2b). However, for the genus *Elatostematoides*, we examined only *Elatostematoides australis* (Wedd.) Yu Hsin Tseng, A.K. Monro, Y.G. Wei & J.M. Hu, which was one of several transferred species from *Elatostema* by Tseng et al. (2019). This species is sister to the remaining *Elatostema* species in our study, as was a clade formed by six *Elatostematoides* species examined by Monro et al. (2025). Hence, both support the recognition of *Elatostematoides* as an independent genus. However, *Elatostematoides* comprises approximately 20 species (Monro et al., 2025), so to thoroughly assess this classification and clarify the relationships among these four genera, significantly expanded taxon sampling will be essential.

#### 4.3.7 Tribe 7. Urticeae (Clade III)

The genus *Laportea* comprises four distinct but unrelated clades (Fig. 2a, Fig. 2b). The first of these (subclade 3C) comprises *Laportea decumana* Wedd., plus *Urticastrum decumanum* Kuntze, supporting the assertion (Chew, 1965, 1969) that they are the same species and hence *Urticastrum* is a synonym of *Laportea*. Our phylogenetic results indicate that this species clusters within another genus *Dendrocnide*. Morphologically, this species differs from other *Laportea* species in its growth habit, leaf form, texture and vestiture, but its stipules link it to *Dendrocnide* (Chew, 1965, 1969). Hence, we strongly recommend transferring this species from *Laportea* to *Dendrocnide*.

The remaining three *Laportea* clades correspond to the three sections recognized by Wang and Chen (1995), based on characteristics of the pedicels and achenes. Subclade 3B comprises the monotypic sect. *Sceptrocnide*, i.e. *Laportea cuspidata* (Wedd.) Friis, which is distinct from other *Laportea* species in having unwinged pedicels of female flowers. Subclade 3E corresponds to sect. *Laportea*, whereas subclade 3F aligns with sect. *Fleurya*. These two sections are noted for their winged pedicels in female flowers, but the former section contains lateral and symmetrical wings, whereas the latter has dorsiventral and asymmetrical ones (Chew, 1969). They also differ in the texture of their achenes, which are either linear or warty in surface depressions, respectively. Therefore, we concur with the proposal by Kim et al. (2015) to elevate *Laportea* sect. *Sceptrocnide* and sect. *Laportea* (as a more narrowly defined *Laportea*, i.e., *Laportea* s.s.) to independent generic rank. To make the latter a monophyletic group requires the separation of sect. *Fleurya* from *Laportea* as a new genus; however, our results did not recover sect. *Fleurya* as monophyletic, because *Laportea ruderalis* (G. Forst.) Chew failed to group with the other members of the section, differing from the results of Kim et al. (2015) and Monro et al. (2025). This discrepancy is likely attributable to the limited chloroplast data retrieved for this species in our study (only 11 plastid genes were successfully extracted) (Table S4). Hence, more extensive sampling and molecular data would be helpful to resolve its taxonomy.

The genus *Urera* is distributed in pantropical regions and comprises *ca*. 35 species, a group that has been repeatedly demonstrated to be non-monophyletic (Friis, 1985; Wells et al., 2021; Ogoma et al., 2022). Our data resolve three clades corresponding to (and hence supporting) its subdivision into three genera by Wells et al. (2021): *Urera* s.s. (Neotropical taxa), *Scepocarpus* (Afrotropic taxa), and an expanded *Touchardia* including all *Urera* species from Hawaii. However, the species *Urera oligoloba* Baker (endemic to Madagascar), falls within none of these clades and instead is sister to *Obetia*. Morphological traits also link it to *Obetia* (e.g., female pedicel with articulation beneath the perianth, cylindrical stigma with a stalk, highly asymmetrical and stipitate achene, reflexed with a hard, sculptured wall) (Friis, 1982). Considering the consistency of evidence from molecular data and morphology, we recommend moving it to *Obetia*. Finally, the genus *Hesperocnide* contains two species: *Hesperocnide sandwicensis* (Wedd.) Wedd. and *Hesperocnide tenella* Torr., which occur in Hawaii and western North America, respectively. Morphologically, the genus *Hesperocnide* shows clear morphological affinities (e.g., opposite leaves, lateral stipules, and straight achenes) with *Urtica* (Wu et al., 2015). In common with Huang et al. (2019) and Monro et al. (2025), our data indicate the type species of *Hesperocnide* (*H*. *tenella*) is nested within *Urtica*, thus supporting its synonymization under *Urtica*.

## 5 Taxonomic treatments

Since the last system of classification of Urticaceae proposed by Friis (1989, 1993b), we have confirmed and updated the intrafamilial framework as outlined above, following the sequence of clades recovered in our phylogenetic tree (Fig. 2a, Fig. 2b). Seven tribes are recognized, comprising one each from Clades II (Elatostemateae) and III (Urticeae), two from the major subclades of Clade IV (Cecropieae and Sarcochlamydeae) and three from the major subclades of Clade I (Boehmerieae, Parietarieae, and Forsskaoleeae). Of these, Sarcochlamydeae *trib*. *et stat*. *nov*. is newly proposed, together with a new genus *Chiajuia gen*. *nov*. The nomenclature of the six previously recognized tribes in Urticaceae follows Conn and Hadiah (2009). The diagnostic descriptions adopted here are predominantly based on Chen et al. (2003) and Friis (1989, 1993b).

1. **Boehmerieae** Gaudich. (1830: 499).

This tribe corresponds to subclades 1A + 1B + 1E within Clade I (Fig. 2a, Fig. 2b). Morphologically, the tribe Boehmerieae comprises herbs, shrubs, and trees. Key characteristics include the presence of punctiform cystoliths; herbaceous, shrubby, or arborescent stems; and leaves that are spirally arranged or opposite, bearing persistent though occasionally early-caducous stipules. The inflorescence is never surrounded by an involucre; perianths are typically connate into a tube or rarely absent, and the stigma is mostly filiform, occasionally capitate-penicillate in the female flowers; the rudimentary ovary is present in male flowers. The achenes are enclosed by dried or fleshy perianths. The tribe includes 14 genera, which are listed alphabetically below.

1.1 ***Archiboehmeria*** C.J. Chen

This genus used to be monotypic, however, phylogenetic evidence demonstrates that *Boehmeria nivea* (L.) Gaudich, an economically important species that has long been recognized as a species of *Boehmeria*, is in fact not closely related to that genus, and instead shows a close relationship with *Archiboehmeria*. Therefore, it is appropriate to expand the circumscription of *Archiboehmeria* to include *B*. *nivea* and its three morphologically distinct varieties (Wu et al., 2024; Zhao et al., 2024, 2025).

***Archiboehmeria nivea*** (L.) ZengY. Wu, X.G. Fu & D.Z. Li, ***comb*. *nov*.**

Basionym: *Urtica nivea* L., Sp. Pl.: 985 (1753). *Boehmeria nivea* (L.) Gaudich., Voy. Uranie: 499 (1830).

(a) ***A*. *nivea*** var. ***nivea***

(b) ***A*. *nivea*** var. ***tenacissima*** (Gaudich.) ZengY. Wu, X.G. Fu & D.Z. Li, ***comb. nov.***

Basionym: *Boehmeria tenacissima* Gaudich., Bot. Freyc. Voy. 500 (1830).

(c) ***A*. *nivea*** var. ***strigosa*** (ZengY. Wu & Y. Zhao) ZengY. Wu, X.G. Fu & D.Z. Li, ***comb. nov.***

Basionym: *Boehmeria nivea* var. *strigosa* ZengY. Wu & Y. Zhao, Guihaia, 44: 1617–1624 (2024).

1.2 ***Astrothalamus*** C.B. Rob.

This genus used to be monotypic, but it shows a close relationship with the newly described Australian species *Debregeasia australis* Friis, Wilmot-Dear & C.J. Chen (Wilmot-Dear and Friis, 2012). However, further investigation, including examination of *D*. *australis* specimens and population sampling, is needed to determine whether *D*. *australis* should be included in *Astrothalamus*.

1.3 ***Boehmeria*** Jacq., Enum. Syst. Pl. 9 (1760). Type: *Boehmeria ramiflora* Jacq. = *Cypholophus* Wedd., Ann. Sci. Nat. Bot. sér. 4, 1: 198 (1854), ***syn***. ***nov***. Lectotype: *Cypholophus macrocephalus* Wedd.

As mentioned above, *Boehmeria nivea* (L.) Gaudich. and *Boehmeria excelsa* Wedd. should be excluded from this genus, while the genus *Cypholophus* should be merged into *Boehmeria*.

1.4 ***Chamabainia*** Wight

1.5 ***Debregeasia*** Gaudich.

*Debregeasia australis* Friis, Wilmot-Dear & C.J. Chen and *Debregeasia wallichiana* Wedd. should be excluded from this genus.

1.6 ***Gonostegia*** Turcz., Bull. Soc. Imp. Naturalistes Moscou 19: 509 (1846). Type: *Gonostegia oppositifolia* Turcz. (designated by Monro et al., 2025).

This genus was synonymized with *Pouzolzia* (sect. *Memorialis*) by Wilmot-Dear and Friis (2004). Our study recommends that *Gonostegia* be maintained as a separate genus, because it occupies an independent phylogenetic position (subclade 1A6). It differs from *Pouzolzia* in having opposite leaves with only basal lateral veins, and is distributed in the tropics and subtropics of Asia and Australia.

1.7 ***Leptocnide*** Blume, Mus. Bot. 2: 193 (1857). Type: *Leptocnide rubricaulis* Blume (≡*Pouzolzia rubricaulis* Wedd.)

This genus is resurrected to comprise *Pouzolzia rubricaulis* Wedd., which markedly differs from all other *Pouzolzia* taxa in its very broad caudate stipules with thick texture, tiny stigma, and very small flower but very large fruit (Kravtsova et al., 2003; Wilmot-Dear and Friis, 2004). The distribution of this genus is restricted to Java and the Philippines.

1.8 ***Margarocarpus*** Wedd., Ann. Sci. Nat. Bot. sér. 4, 1: 203 (1854). Lectotype: *Margarocarpus vimineus* Wedd.

This genus is resurrected to comprise *Pouzolzia sanguinea* (Blume) Merr. (including the synonymized *P*. *calophylla* W.T. Wang & C.J. Chen). This species forms an isolated monophyletic clade in the phylogeny (Fig. 2a, Fig. 2b). Morphologically, *P. sanguinea* is often a tree with chartaceous or coriaceous bicolored leaves, and vegetatively very variable with a widespread distribution in Asian-Malesian regions (Wilmot-Dear and Friis, 2004; Monro et al., 2025).

1.9 ***Neraudia*** Gaudich.

1.10 ***Nothocnide*** Blume

1.11 ***Oreocnide*** Miq.

1.12 ***Phenax*** Wedd.

*Phenax madagascariensis* Leandri should be excluded from this genus.

1.13 ***Pipturus*** Wedd.

1.14 ***Pouzolzia*** Gaudich.

The genus *Pouzolzia* is accepted, with *Hemistylus*, *Neodistemon*, and *Rousselia* treated as its synonyms. Considering the morphological studies of Friis (1989, 1993a, 1993b) along with our molecular phylogenetic results, *Phenax madagascariensis* Leandri should be a member of this genus. Meanwhile, *Pouzolzia australis* (Endl.) Friis & Wilmot-Dear, *P*. *niveotomentosa* W.T. Wang, *P*. *rubricaulis* Wedd., and *P*. *sanguinea* (Blume) Merr. should be excluded from this genus. We propose the following new combination.

***Pouzolzia madagascariensis*** (Leandri) ZengY. Wu, X.G. Fu & D.Z. Li, ***comb*. *nov***.

Basionym: *Phenax madagascariensis* Leandri, Ann. Mus. Colon. Marseille, sér. 6, 7–8: 68 (1950).

2. **Parietarieae** Gaudich. (1830: 511).

This tribe corresponds to subclade 1C within Clade I (Fig. 2a, Fig. 2b). Morphologically, the tribe Parietarieae comprises creeping herbs and spreading shrubs or small trees. Key characteristics include the presence of punctiform cystoliths; herbaceous, shrubby, or arborescent stems; and leaves that are spirally arranged without stipules. The inflorescence is often surrounded by an involucre; perianths are consistently present with 3 or 4 lobes, and the stigma is linear or penicillate in the female flowers; the rudimentary ovary is present in male flowers. The achenes are enclosed by perianths and involucre. The tribe includes three genera, listed alphabetically below.

2.1 ***Gesnouinia*** Gaudich.

2.2 ***Parietaria*** L.

The genus *Parietaria* does not form a monophyletic group but instead comprises two clades corresponding to annual and perennial species (Fig. 3). Further sampling within *Parietaria* is needed to verify whether the genus should be split, or whether *Gesnouinia* and *Soleirolia* should be merged within this genus.

2.3 ***Soleirolia*** Gaudich.

3. **Forsskaoleeae** Gaudich. (1830: 544).

This tribe corresponds to subclade 1D within Clade I (Fig. 2a, Fig. 2b). Morphologically, the tribe Forsskaoleeae comprises herbs and rarely shrubs or small trees. Key characteristics include the presence of punctiform cystoliths; herbaceous, shrubby, or arborescent stems; leaves that are alternate or opposite with stipules. The inflorescence is often surrounded by an involucre; perianths are pouchlike or absent, and the stigma is filiform in the female flowers; the male flowers are boat-shaped and bear a single stamen (a feature unique among the tribes of Urticaceae); and they lack a rudimentary ovary. The achenes are enclosed by perianths and involucre. The tribe includes five genera, listed alphabetically below.

3.1 ***Australina*** Gaudich.

3.2 ***Didymodoxa*** E. Mey. ex Wedd.

3.3 ***Droguetia*** Gaudich.

3.4 ***Forsskaolea*** L.

3.5 ***Metatrophis*** F. Br.

This monotypic genus is endemic to Rapa Island, French Polynesia. Our phylogenetic analyses revealed that *Metatrophis* belongs to the tribe Forsskaoleeae. Investigation of the protologue and local floras further indicates that its morphology is entirely consistent with the diagnostic features of Forsskaoleeae.

4. **Cecropieae** Gaudich. (1830: 506).

This tribe corresponds to subclade 4A within Clade IV (Fig. 2a, Fig. 2b). Morphologically, the tribe Cecropieae comprises shrubs and trees. Key characteristics include the absence of cystoliths; shrubby or arborescent stems; leaves that are spirally arranged; entire to (sub)palmate; and stipules that are usually large. The inflorescence is often repeatedly branched and sometimes surrounded by a caducous spathe; perianths are tubular, and the stigma is capitate-penicillate or lingulate in the female flowers; the male flowers have straight filaments but lack a rudimentary ovary. The achenes are often enclosed by succulent perianths. The tribe includes six genera, listed alphabetically below.

4.1 ***Cecropia*** Loefl.

**Fig. 5:**
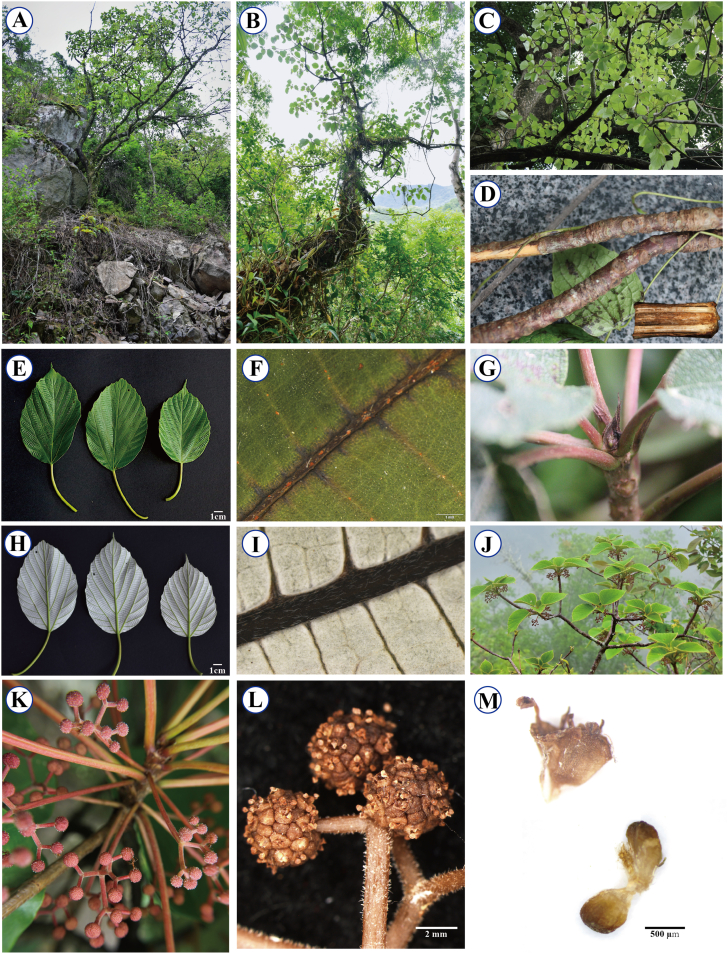
Photos of living plants of *Chiajuiawallichiana* (Wedd.) ZengY.Wu, X.G.Fu & D.Z.Li. A, habitat; B, habit; C, spreading branches; D, leaf scars and longitudinal section of the stem (lower right); E–F, adaxial surface of leaf blade; G, stipules; H–I, abaxial surface of leaf blade; J, arrangement of leaves and inflorescences; K, inflorescence; L, stamens and pedicel; M, staminate (upper left) and pistillate flowers (lower right). All photos were taken by Li-Juan Deng and Yin-Lei Li, except for K (by Sam Kuzhalanattu), and are used with permission.

4.2 ***Chiajuia*** ZengY. Wu, X.G. Fu & D.Z. Li, ***gen*. *nov*.** 家瑞麻属 (新拟). Type: *Chiajuia wallichiana* (Wedd.) ZengY. Wu, X.G. Fu & D.Z. Li, ***comb***. ***nov*.** – Fig. 5.

Basionym: *Debregeasia wallichiana* Wedd., Arch. Mus. Hist. Nat. 464 (1857).

Diagnosis: Shrubs or small trees habit to 6 m; stems shrubby or arborescent, and stout; spirally arranged leaves crowded towards the apex with very large leaf-scars and stipules, 12–20 × 2.5–5 mm. The inflorescence is borne on current and previous years’ branches, and is 3–7-dichotomously branched, length 3.5–7.5 × 3–6.5 cm; peduncle 2.5–6.5 cm, indumentum spreading hirtellous; glomerules globose, 3–5 mm in diam. Female flowers sessile, obovoid, *ca*. 0.7–0.8 mm; perianth tube membranous, 4-ribbed, 4-denticulate at apex. Male flowers shortly pedicellate, obovoid in bud 1 mm in diam., with straight filaments; perianth lobes 5, broadly ovate, glabrous abaxially, connate at the middle, apex acute; rudimentary ovary stipitate, obovoid, ca. 0.6 mm. Achenes *ca*. 1.3–1.5 mm, enclosed by membranous perianth but not adnate to it (Chen et al., 2003). This is a monotypic genus.

Etymology: The genus is named in honor of Prof. Chia-Jui Chen (affiliated with the herbarium of Institute of Botany, Chinese Academy of Sciences, PE), a distinguished specialist of Urticaceae, for his significant contributions to the taxonomy of the family in Asia.

Distribution: Southern Yunnan, China and the Indian Subcontinent to Mainland Southeast Asia

Habitat: Limestone forest

Phenology: Flowers from May to August, and fruits from July to September.

4.3 ***Coussapoa*** Aubl.

4.4 ***Musanga*** C. Sm. ex R. Br.

4.5 ***Myrianthus*** P. Beauv.

4.6 ***Pourouma*** Aubl.

5. **Sarcochlamydeae** (Wedd.) ZengY. Wu, X.G. Fu & D.Z. Li, ***trib.*** ***et*** ***stat. nov.*** 肉被麻族 (新拟). Type: *Sarcochlamys* Gaudich.

Basionym: subtribe Sarcochlamydinae Wedd., Ann. Sci. Nat., Bot., sér. 4, 1: 175 (1854).

Diagnosis: Shrubs or small trees, without stinging hairs; cystoliths punctiform (often difficult to observe under a light microscope); stems shrubby or arborescent; leaves alternate; stipules deciduous, intrapetiolar, 2-lobed or entire; leaf blade 3-veined, margin serrate, often tomentose below. Inflorescences axillary cymes. Flowers unisexual (plants monoecious or dioecious). Female flowers: perianth cupular, adnate at the base of the ovary; ovule straight; stigma sessile, penicillate or ringlike; staminodes absent. Male flowers: perianth lobes 4 or 5, segments valvate or imbricate; stamens 4 or 5; rudimentary ovary present, often lanate. The fruit type is achene. Seed with thin endosperm (Friis, 1993b; Chen et al., 2003). This new tribe occurs in tropical and subtropical regions of Asia and the South Pacific. It comprises four genera.

5.1 ***Gibbsia*** Rendle

This genus comprises two species: *Gibbsia carstenszensis* Rendle and *G*. *insignis* Rendle (Friis, 1993b). *G*. *insignis* is nested within *Maoutia* in our phylogenetic analyses, so *Gibbsia* may be best synonymized with *Maoutia*. However, the other recognized *Gibbsia* species must first be examined before a taxonomic decision is made.

5.2 ***Leucosyke*** Zoll. & Moritzi

5.3 ***Maoutia*** Wedd.

5.4 ***Sarcochlamys*** Gaudich.

6. **Elatostemateae** Gaudich. (1830: 493).

This tribe corresponds to Clade II (Fig. 2a, Fig. 2b). Morphologically, the tribe Elatostemateae comprises herbs (usually succulent), shrubs and trees. Key characteristics include the linear cystoliths; herbaceous, shrubby, or arborescent stems; and leaves that are spirally arranged or opposite, bearing well developed though sometimes early-caducous stipules. The inflorescence is axillary, rarely branched, and sometimes seated on a receptacle with a few bracteoles; the female flowers have 3–5 perianths lobes with inflexed staminodes, and the stigma is penicillate; the male flowers have a very small rudimentary ovary. The achenes are partly enclosed by perianths. The tribe includes ten genera, listed alphabetically below.

6.1 ***Achudemia*** Blume

6.2 ***Elatostema*** J.R. Forst. & G. Forst., Char. Gen. Pl.: 105 (1776), nom. cons. Type: *Elatostema sessile* J.R. Forst. & G. Forst. = *Pellionia* Gaudich., Voy. Uranie: 499 (1830), nom. cons., ***syn***. ***nov***. Type: *Pellionia elatostemoides* Gaudich.

6.3 ***Elatostematoides*** C.B. Rob., Philipp. J. Sci., C 5: 497 (1911). Type: *Elatostematoides manillensis* (Wedd.) C.B. Rob. (‘*manillense*’) (*Elatostema manillense* Wedd.)

This genus was resurrected from a subgenus of *Elatostema* by Tseng et al. (2019), and can be morphologically identified as shrub with inflorescence having neither an involucre nor conspicuous tepals in female flowers. Consistent with Tseng et al. (2019) and Monro et al. (2025), our study indicates that it is sister to *Elatostema* (including *Pellionia*). Therefore, we support the recognition of *Elatostematoides* as a distinct genus. However, since only one species of *Elatostematoides* was sampled in this study, and given the considerable overlap in morphological characters between *Elatostematoides* and *Pellionia*, the taxonomic treatment still requires further investigation. The distribution of this genus is restricted to Southeast Asia and Pacific Islands.

6.4 ***Gyrotaenia*** Griseb.

This genus, as currently defined, is non-monophyletic. It is closely related to *Myriocarpa* based on plastome data, while *Gyrotaenia* species occur intermixed with *Myriocarpa* in our nrDNA tree. Both genera are restricted to South Tropical America, and they share many overlapping morphologies, such as growth form, phyllotaxis, and a dioecious mating system (Friis, 1993b; Wu et al., 2015). Further study using more nuclear genes and species of *Gyrotaenia* and *Myriocarpa* is needed to clarify their relationships.

6.5 ***Lecanthus*** Wedd.

6.6 ***Metapilea*** W.T. Wang

6.7 ***Myriocarpa*** Benth.

6.8 ***Petelotiella*** Gagnep.

6.9 ***Pilea*** Lindl., Coll. Bot. ad. t. 4 (1821), nom. cons. Type: *Pilea muscosa* Lindl., nom. illeg. (*Parietaria microphylla* L., *Pilea microphylla* (L.) Liebm.) = *Haroldiella* J. Florence, Fl. Polynésie Fr. 1: 218 (1997), ***syn***. ***nov***. Type: *Haroldiella sykesii* J. Florence.

6.10 ***Procris*** Comm. ex Juss.

This genus also includes *Pellionia repens* (Lour.) Merr, which was previously treated as *Procris repens* (Lour.) B.J. Conn & Hadiah by Conn and Hadiah (2011).

7. **Urticeae** Lam. & DC. (1806: 184)

This tribe corresponds to Clade III (Fig. 2a, Fig. 2b). Morphologically, the tribe Urticeae comprises herbs, shrubs and trees. Key characteristics include the punctiform cystoliths; the presence of stinging hairs (a feature unique among the tribes of Urticaceae); herbaceous, shrubby, or arborescent stems; leaves that are spirally arranged or opposite, bearing well developed though sometimes early-caducous stipules. The inflorescence is axillary, pedunculate, and often forms irregularly branched panicles; the female flowers have 4 perianths lobes without staminodes, and the stigma is filiform, ligulate, or capitate-penicillate; the male flowers have a rudimentary ovary. The achenes are enclosed by fleshy or membranous perianths. The tribe includes 13 genera, listed alphabetically below.

7.1 ***Dendrocnide*** Miq.

This genus should be circumscribed to include *Laportea decumana* Wedd. Morphologically, this species is distinguished from other *Laportea* species by its growth habit, leaf morphology, texture, and vestiture, while its stipular characteristics and phylogenetic affinities align it with *Dendrocnide* (Chew, 1965, 1969). Therefore, we strongly recommend the transfer of this species from *Laportea* to *Dendrocnide*. Accordingly, we propose the following new combination.

***Dendrocnide decumana*** (Wedd.) ZengY. Wu, X.G. Fu & D.Z. Li, ***comb*. *nov***.

Basionym: *Laportea decumana* Wedd., Nouv. Arch. Mus. Hist. Nat. 9: 129 (1856).

7.2 ***Discocnide*** Chew

7.3 ***Girardinia*** Gaudich.

7.4 ***Laportea*** Gaudich.

The genus *Laportea* is accepted, with *Urticastrum* treated as a rejected name. This genus should only comprise species of *Laportea* sect. *Laportea* (subclade 3E, Fig. 2a, Fig. 2b), as recognized by Wang and Chen (1995). Therefore, species from *Laportea* sect. *Sceptrocnide* and sect. *Fleurya* should be excluded from *Laportea*. However, the monophyly of *Laportea* sect. *Fleurya* is poorly supported in our current study, hence we do not recognize it as a distinct genus at present and recommend more extensive sampling and molecular data to resolve its taxonomy. Hence species of sect. *Fleurya* should temporarily remain members of *Laportea* until this is resolved.

7.5 ***Nanocnide*** Blume

7.6 ***Obetia*** Gaudich.

The species *Urera oligoloba* Baker (endemic to Madagascar) falls into none of the *Urera* clades and instead is sister to *Obetia*. Morphological traits also link it to *Obetia* (e.g., female pedicel with articulation beneath the perianth, cylindrical stigma with a stalk, and a highly asymmetrical and stipitate achene, reflexed with a hard, sculptured wall) (Friis, 1982). Considering the consistency of evidence from molecular data and morphology, we recommend moving it to *Obetia*. Therefore, based on the basionym *Urera oligoloba*, we propose the following new combination.

***Obetia oligoloba*** (Baker) ZengY. Wu, X.G. Fu & D.Z. Li, ***comb*. *nov***.

Basionym: *Urera oligoloba* Baker, J. Linn. Soc., Bot. 20: 265 (1883).

7.7 ***Poikilospermum*** Zipp. ex Miq.

7.8 ***Scepocarpus*** Wedd., A.P. de Candolle, Prodr. 16: 98 (1869). Type: *Scepocarpus mannii* Wedd.

This genus was resurrected from the previously synonymized *Scepocarpus* Wedd. to accommodate Afrotropical species formerly placed in *Urera*. Morphologically, *Scepocarpus* species are lianas, characterized by relatively uniform leaf morphology with few exceptions, and a largely fused perianth in female flowers (Friis, 1985). A detailed taxonomic treatment for the species has been provided by Wells et al. (2021).

7.9 ***Sceptrocnide*** Maxim. Bull. Acad. Imp. Sci. Saint-Pétersbourg, sér. 3, 22: 238 (1876). Type: *Sceptrocnide macrostachya* Maxim.

This genus was resurrected from the monotypic sect. *Sceptrocnide* of *Laportea*, i.e. the species *Laportea cuspidata* (Wedd.) Friis treated by Wang and Chen (1995). Morphologically, it is distinct from other *Laportea* species in having unwinged pedicels of female flowers. Consistent with Kim et al. (2015) and Monro et al. (2025), our study indicates that it is sister to *Nanocnide*. Therefore, we support the recognition of *Sceptrocnide* as a distinct genus and propose the following new combination. This genus is native to China, Japan, Korea, and Myanmar.

***Sceptrocnide cuspidata*** (Wedd.) ZengY. Wu, X.G. Fu & D.Z. Li, ***comb*. *nov***.

Basionym: *Girardinia cuspidata* Wedd., A.P. de Candolle, Prodr. 16: 103 (1869).

7.10 ***Touchardia*** Gaudich.

This genus also includes Hawaiian *Urera* species previously assigned to *Touchardia* by Wells et al. (2021).

7.11 ***Urera*** Gaudich.

This genus should only comprise Neotropical species. Morphologically, they are erect shrubs or small trees, having rather varied leaf morphologies and a 4-lobed female perianth (Friis, 1985). Therefore, species from Afrotropic regions and Hawaii should be excluded from *Urera*.

7.12 ***Urtica*** L.

The genus *Urtica* is accepted, with *Hesperocnide* treated as its synonym. Therefore, based on the basionym *Hesperocnide tenella* Torr., we propose the following new combination.

***Urtica tenella*** (Torr.) ZengY. Wu, X.G. Fu & D.Z. Li, ***comb*. *nov***.

Basionym: *Hesperocnide tenella* Torr., Pacif. Railr. Rep. Whipple, Bot. 4: 139 (1857).

7.13 ***Zhengyia*** T. Deng, D.G. Zhang & H. Sun.

## 6 Conclusions

This study demonstrates that extensive sampling of both the genome and taxa can provide new phylogenetic insights, successfully elucidating both deep and shallow relationships within Urticaceae. Notably, many of the newly resolved groups are supported by morphological traits. The four clades comprising Urticaceae (I–IV) are robustly supported. To the six recognized tribes (Boehmerieae, Cecropieae, Elatostemateae, Forsskaoleeae, Parietarieae, and Urticeae), we added a seventh, Sarcochlamydeae, which is sister to Cecropieae in Clade IV. In addition, we describe one new genus, *Chiajuia* within Cecropieae.

Although our sampling adequately elucidates nearly all intergeneric relationships within this family, further research is needed to add genera that we were unable to collect here (e.g., *Achudemia*, *Metapilea*, *Petelotiella*), and examine more species for certain genera (e.g., *Elatostema*, *Elatostematoides*, *Gibbsia*, *Gyrotaenia*, *Laportea*, *Myriocarpa*, *Parietaria*, *Pellionia*, and *Urera*). In addition, nuclear markers should be explored to deepen our understanding of phylogenetic relationships within Urticaceae. Furthermore, this study involves several species that are presently unassigned to any genus, including *Boehmeria excelsa*, *Pouzolzia australis*, and *Pouzolzia niveotomentosa*, whose taxonomic status needs to be further clarified in future work. In any case, the phylogenomic framework and updated classification of Urticaceae provided here are poised to serve as a robust framework for future ecological and evolutionary inquiries on this family.

## CRediT authorship contribution statement

**Xiao-Gang Fu:** Data curation, Formal analysis, Software, Visualization, Investigation, Writing – original draft, Writing – review & editing. **Jie Liu:** Resources, Conceptualization, Supervision, Project administration, Funding acquisition, Visualization, Writing – review & editing. **Richard I. Milne:** Resources, Conceptualization, Supervision, Writing – review & editing. **Alex K. Monro:** Resources, Supervision, Writing – review & editing. **Shui-Yin Liu:** Writing – review & editing. **Qin Tian:** Writing – review & editing. **Gregory W. Stull:** Writing – review & editing. **Amos Kipkoech:** Writing – review & editing. **Ting-Shuang Yi:** Resources, Conceptualization, Supervision, Project administration, Funding acquisition, Writing – review & editing. **De-Zhu Li**: Resources, Conceptualization, Supervision, Project administration, Funding acquisition, Writing – review & editing. **Zeng-Yuan Wu**: Resources, Conceptualization, Supervision, Project administration, Funding acquisition, Writing – review & editing.

## Data availability

The raw genome skimming data used in this study have been deposited in the Genome Sequence Archive (GSA: CRA032101) at the National Genomics Data Center, China National Center for Bioinformation/Beijing Institute of Genomics, Chinese Academy of Sciences, under accession numbers CRX2091429–CRX2091839 (Table S2), and are publicly accessible at https://ngdc.cncb.ac.cn/gsa. All gene sequences with their final alignments, and the resulting phylogenetic tree files that support the findings of this study are openly available in the Science Data Bank at https://www.scidb.cn/doi/10.6084/m9.figshare.30438233.

## Declaration of competing interest

The authors declare that they have no known competing financial interests or personal relationships that could have appeared to influence the work reported in this paper.

## References

[bib1] Baker W.J., Bailey P., Barber V. (2022). A comprehensive phylogenomic platform for exploring the angiosperm tree of life. Syst. Biol..

[bib2] Bankevich A., Nurk S., Antipov D. (2012). SPAdes: a new genome assembly algorithm and its applications to single-cell sequencing. J. Comput. Biol..

[bib3] Berg C.C. (1978). Cecropiaceae a new family of the Urticales. Taxon.

[bib4] Birky C.W. (1995). Uniparental inheritance of mitochondrial and chloroplast genes: mechanisms and evolution. Proc. Natl. Acad. Sci. U.S.A..

[bib5] Bolger A.M., Lohse M., Usadel B. (2014). Trimmomatic: a flexible trimmer for illumina sequence data. Bioinformatics.

[bib6] Borowiec M.L. (2016). AMAS: a fast tool for alignment manipulation and computing of summary statistics. PeerJ.

[bib7] Borowiec M.L. (2019). Spruceup: fast and flexible identification, visualization, and removal of outliers from large multiple sequence alignments. J. Open Source Softw..

[bib8] Brown F.B.H., Brown F.B.H. (1935). Flora of Southeastern Polynesia, III: Dicotyledons.

[bib9] Brown J.W., Walker J.F., Smith S.A. (2017). Phyx: phylogenetic tools for unix. Bioinformatics.

[bib10] Cai L.M., Zhang H.R., Davis C.C. (2022). PhyloHerb: a high-throughput phylogenomic pipeline for processing genome-skimming data. Appl. Plant Sci..

[bib11] Chen C.J. (1980). *Archiboehmeria* C.J. Chen – a new genus of Urticaceae. Acta Phytotaxon. Sin..

[bib12] Chen C.J. (1985). *Sphaerotylos* C.J. Chen – a remarkable new genus of Urticaceae from China with notes on stigmas of the family. Acta Phytotaxon. Sin..

[bib13] Chen C.J., Lin Q., Friis I., Wu Z.Y., Raven P.H. (2003). Flora of China.

[bib14] Chew W.L. (1965). *Laportea* and allied genera (Urticaceae). Gard. Bull. (Singap.).

[bib15] Chew W.L. (1969). A monograph of *Laportea* (Urticaceae). Gard. Bull. (Singap.).

[bib16] Conn B.J., Hadiah J.T. (2009). Nomenclature of tribes within the Urticaceae. Kew Bull..

[bib17] Conn B.J., Hadiah J.T. (2011). Precursor to flora account of *Procris* (Urticaceae) in Peninsular Malaysia. Gard. Bull. (Singap.).

[bib18] Corriveau J.L., Coleman A.W. (1988). Rapid screening method to detect potential biparental inheritance of plastid DNA and results for over 200 angiosperm species. Am. J. Bot..

[bib19] de Jussieu, A.L., 1789. Genera Plantarum Secundum Ordines Naturales Disposita, Juxta Methodum in Horto Regio Parisiensi Exaratam, Anno MDCCLXXIV. Herissant, Paris.

[bib20] Delsuc F., Brinkmann H., Philippe H. (2005). Phylogenomics and the reconstruction of the tree of life. Nat. Rev. Genet..

[bib21] Deng T., Kim C., Zhang D.G. (2013). *Zhengyia shennongensis*: a new bulbiliferous genus and species of the nettle family (Urticaceae) from central China exhibiting parallel evolution of the bulbil trait. Taxon.

[bib22] Dhouibi R., Affes H., Salem Ben (2020). Screening of pharmacological uses of *Urtica dioica* and others benefits. Prog. Biophys. Mol. Biol..

[bib23] Doukkali Z., Taghzouti K., Bouidida E.H. (2015). Evaluation of anxiolytic activity of methanolic extract of *Urtica urens* in a mice model. Behav. Brain Funct..

[bib24] Doyle J.J., Doyle J.L. (1987). A rapid DNA isolation procedure for small quantities of fresh leaf material. Phytochem. Bull..

[bib25] Florence J. (1997).

[bib26] Friis I. (1982). The identity of *Urera longifolia* and *U. oligoloba* – a supplement to Chew's monograph of *Laportea* (Urticaceae). Nord. J. Bot..

[bib27] Friis I. (1985). The genus *Urera* (Urticaceae) in eastern tropical Africa. Nord. J. Bot..

[bib28] Friis I., Crane P.R., Blackmore S. (1989). Evolution, Systematics, and Fossil History of the Hamamelidae.

[bib29] Friis I. (1993). The distribution of *Phenax sonneratii* and the identity of *Pouzolzia conulifera* (Urticaceae). Kew Bull..

[bib30] Friis I., Kubitzki K., Rohwer J.G., Bittrich V. (1993). The Families and Genera of Vascular Plants, Flowering Plants: Dicotyledones.

[bib31] Friis I., Wilmot-Dear C.M. (1988). A revision of the tribe Forsskaoleae (Urticaceae). Nord. J. Bot..

[bib32] Fu L.F., Monro A.K., Yang T.G. (2021). *Elatostema qinzhouense* (Urticaceae), a new species from limestone karst in Guangxi, China. PeerJ.

[bib33] Fu L.F., Wen F., Maurin O. (2022). A revised delimitation of the species-rich genus *Pilea* (Urticaceae) supports the resurrection of *Achudemia* and a new infrageneric classification. Taxon.

[bib34] Fu L.F., Xiong C., Monro A.K. (2022). *Pilea danxiaensis* (Urticaceae), a new species in the Danxia landform from Guangdong, China including a description of the entire chloroplast genome. PhytoKeys.

[bib35] Gardner E.M., Bruun-Lund S., Niissalo M. (2023). Echoes of ancient introgression punctuate stable genomic lineages in the evolution of figs. Proc. Natl. Acad. Sci. U.S.A..

[bib36] Gaudichaud C., Freycinet H.d. (1830). Voyage autour du monde.

[bib37] Gonçalves D.J.P., Simpson B.B., Ortiz E.M. (2019). Incongruence between gene trees and species trees and phylogenetic signal variation in plastid genes. Mol. Phylogenet. Evol..

[bib38] Gu W., Zhang T., Liu S.Y. (2024). Phylogenomics, reticulation, and biogeographical history of Elaeagnaceae. Plant Divers..

[bib39] Guo C., Luo Y., Gao L.M. (2023). Phylogenomics and the flowering plant tree of life. J. Integr. Plant Biol..

[bib40] Hadiah J.T., Conn B.J., Quinn C.J. (2008). Infra-familial phylogeny of Urticaceae, using chloroplast sequence data. Aust. Syst. Bot..

[bib41] Hoang D.T., Chernomor O., von Haeseler A. (2018). UFBoot2: improving the ultrafast bootstrap approximation. Mol. Biol. Evol..

[bib42] Hou Z., Ma X., Shi X. (2022). Phylotranscriptomic insights into a Mesoproterozoic-Neoproterozoic origin and early radiation of green seaweeds (Ulvophyceae). Nat. Commun..

[bib43] Huang X.H., Deng T., Moore M.J. (2019). Tropical Asian origin, boreotropical migration and long-distance dispersal in nettles (Urticeae, Urticaceae). Mol. Phylogenet. Evol..

[bib44] Jestrow B., Valdés J.J., Jiménez-Rodríguez F. (2012). Phylogenetic placement of the Dominican Republic endemic genus *Sarcopilea* (Urticaceae). Taxon.

[bib45] Jin J.J., Yu W.B., Yang J.B. (2020). GetOrganelle: a fast and versatile toolkit for accurate de novo assembly of organelle genomes. Genome Biol..

[bib46] Johnson M.G., Gardner E.M., Liu Y. (2016). HybPiper: extracting coding sequence and introns for phylogenetics from high-throughput sequencing reads using target enrichment. Appl. Plant Sci..

[bib47] Junier T., Zdobnov E.M. (2010). The Newick utilities: high-throughput phylogenetic tree processing in the Unix shell. Bioinformatics.

[bib48] Kalyaanamoorthy S., Minh B.Q., Wong T.K.F. (2017). ModelFinder: fast model selection for accurate phylogenetic estimates. Nat. Methods.

[bib49] Katoh K., Standley D.M. (2013). MAFFT multiple sequence alignment software version 7: improvements in performance and usability. Mol. Biol. Evol..

[bib50] Kearse M., Moir R., Wilson A. (2012). Geneious basic: an integrated and extendable desktop software platform for the organization and analysis of sequence data. Bioinformatics.

[bib51] Kim C., Deng T., Chase M.W. (2015). Generic phylogeny and character evolution in Urticeae (Urticaceae) inferred from nuclear and plastid DNA regions. Taxon.

[bib52] Kipkoech A., Li K., Milne R.I. (2025). An integrative approach clarifies species delimitation and biogeographic history of *Debregeasia* (Urticaceae). Plant Divers..

[bib53] Koenen E.J.M., Ojeda D.I., Steeves R. (2020). Large-scale genomic sequence data resolve the deepest divergences in the legume phylogeny and support a near-simultaneous evolutionary origin of all six subfamilies. New Phytol..

[bib54] Kravtsova T.I. (2007). A system of the family Urticaceae. Bot. Zhurn. (St. Petersburg).

[bib55] Kravtsova T.I., Tzvelev N.N., Vassilyev A.E. (2009). Comparative Carpology of the Urticaceae Juss.

[bib56] Kravtsova T.I., Friis I., Wilmot-Dear C.M. (2003). Morphology and anatomy of fruits in *Pouzolzia* (Urticaceae) in relation to taxonomy. Kew Bull..

[bib57] Lanfear R., Frandsen P.B., Wright A.M. (2017). PartitionFinder 2: new methods for selecting partitioned models of evolution for molecular and morphological phylogenetic analyses. Mol. Biol. Evol..

[bib58] Lanzilao G., Goswami P., Blackburn R.S. (2016). Study of the morphological characteristics and physical properties of Himalayan giant nettle (*Girardinia diversifolia* L.) fibre in comparison with European nettle (*Urtica dioica* L.) fibre. Mater. Lett..

[bib59] Li H.T., Luo Y., Gan L. (2021). Plastid phylogenomic insights into relationships of all flowering plant families. BMC Biology.

[bib60] Li H., Durbin R. (2009). Fast and accurate short read alignment with Burrows–wheeler transform. Bioinformatics.

[bib61] Mabberley D.J., Mabberley D.J. (2017). Mabberley's plant-book.

[bib62] Martin W., Deusch O., Stawski N. (2005). Chloroplast genome phylogenetics: why we need independent approaches to plant molecular evolution. Trends Plant Sci..

[bib63] Monro A.K. (2006). The revision of species-rich genera: a phylogenetic framework for the strategic revision of *Pilea* (Urticaceae) based on cpDNA, nrDNA, and morphology. Am. J. Bot..

[bib64] Monro A.K., Maurin O., Fu L.F. (2025). Classification of Urticaceae based on morphology and phylogenetic inference. bioRxiv.

[bib65] Monro A.K., Wei Y.G., Chen C.J. (2012). Three new species of *Pilea* (Urticaceae) from limestone karst in China. PhytoKeys.

[bib66] Morales-Briones D.F., Lin N., Huang E.Y. (2022). Phylogenomic analyses in Phrymaceae reveal extensive gene tree discordance in relationships among major clades. Am. J. Bot..

[bib67] Nguyen L.T., Schmidt H.A., Von Haeseler A. (2015). IQ-TREE: a fast and effective stochastic algorithm for estimating maximum-likelihood phylogenies. Mol. Biol. Evol..

[bib68] Nie Z.L., Hodel R., Ma Z.Y. (2023). Climate-influenced boreotropical survival and rampant introgressions explain the thriving of new world grapes in the north temperate zone. J. Integr. Plant Biol..

[bib69] Ogoma C.A., Liu J., Stull G.W. (2022). Deep insights into the plastome evolution and phylogenetic relationships of the tribe Urticeae (Family Urticaceae). Front. Plant Sci..

[bib70] Pardo-De La Hoz C.J., Magain N., Piatkowski B. (2023). Ancient rapid radiation explains most conflicts among gene trees and well-supported phylogenomic trees of nostocalean Cyanobacteria. Syst. Biol..

[bib71] Pyron R.A. (2015). Post-molecular systematics and the future of phylogenetics. Trends Ecol. Evol..

[bib72] Revell L.J. (2012). Phytools: an R package for phylogenetic comparative biology (and other things). Methods Ecol. Evol..

[bib73] Rose J.P., Toledo C.A.P., Lemmon E.M. (2021). Out of sight, out of mind: widespread nuclear and plastid-nuclear discordance in the flowering plant genus *Polemonium* (Polemoniaceae) suggests widespread historical gene flow despite limited nuclear signal. Syst. Biol..

[bib74] Sayyari E., Mirarab S. (2016). Fast coalescent-based computation of local branch support from quartet frequencies. Mol. Biol. Evol..

[bib75] Schüßler C., Bräuchler C., Reyes-Betancort J.A. (2019). Island biogeography of the Macaronesian *Gesnouinia* and Mediterranean *Soleirolia* (Parietarieae, Urticaceae) with implications for the evolution of insular woodiness. Taxon.

[bib76] Slater G.S.C., Birney E. (2005). Automated generation of heuristics for biological sequence comparison. BMC Bioinformatics.

[bib77] Smith S.A., Moore M.J., Brown J.W. (2015). Analysis of phylogenomic datasets reveals conflict, concordance, and gene duplications with examples from animals and plants. BMC Evol. Biol..

[bib78] Smith S.D., Pennell M.W., Dunn C.W. (2020). Phylogenetics is the new genetics (for most of biodiversity). Trends Ecol. Evol..

[bib79] Soltis E.D., Soltis P.S. (2000). Contributions of plant molecular systematics to studies of molecular evolution. Plant Mol. Biol..

[bib80] Soto Gomez M., Pokorny L., Kantar M.B. (2019). A customized nuclear target enrichment approach for developing a phylogenomic baseline for *Dioscorea* yams (Dioscoreaceae). Appl. Plant Sci..

[bib81] Staats M., Erkens R.H.J., van de Vossenberg (2013). Genomic treasure troves: complete genome sequencing of herbarium and insect museum specimens. PLoS One.

[bib82] Stamatakis A. (2014). RAxML version 8: a tool for phylogenetic analysis and post-analysis of large phylogenies. Bioinformatics.

[bib83] Stevens P.F. (2017). http://www.mobot.org/MOBOT/research/APweb/.

[bib84] Subedee B.R., Chaudhary R.P., Uprety Y. (2020). Socio-ecological perspectives of Himalayan giant nettle (*Girardinia diversifolia* (Link) Friis) in Nepal. J. Nat. Fibers.

[bib85] Tian Q., Stull G.W., Kellermann J. (2024). Rapid *in situ* diversification rates in Rhamnaceae explain the parallel evolution of high diversity in temperate biomes from global to local scales. New Phytol..

[bib86] Treiber E.L., Gaglioti A.L., Romaniuc-Neto S. (2016). Phylogeny of the Cecropieae (Urticaceae) and the evolution of an ant-plant mutualism. Syst. Bot..

[bib87] Tseng Y.H., Monro A.K., Wei Y.G. (2019). Molecular phylogeny and morphology of *Elatostema* s.l. (Urticaceae): implications for inter- and infrageneric classifications. Mol. Phylogenet. Evol..

[bib88] Twyford A.D., Ness R.W. (2017). Strategies for complete plastid genome sequencing. Mol. Ecol. Resour..

[bib89] Villaverde T., Larridon I., Shah T. (2023). Phylogenomics sheds new light on the drivers behind a long-lasting systematic riddle: the figwort family Scrophulariaceae. New Phytol..

[bib90] Walker J.F., Walker-Hale N., Vargas O.M. (2019). Characterizing gene tree conflict in plastome-inferred phylogenies. PeerJ.

[bib91] Wang R.N., Milne R.I., Du X.Y. (2020). Characteristics and mutational hotspots of plastomes in *Debregeasia* (Urticaceae). Front. Genet..

[bib92] Wang W.T. (2014).

[bib93] Wang W.T. (2016). Two new species of Urticaceae from China. Bull. Bot. Res..

[bib94] Wang W.T., Chen C.J. (1995).

[bib95] Wang W.T., Wu Z.Y. (2016). Six new species of *Elatostema* (Urticaceae) from Yunnan. Plant Divers..

[bib96] Weddell H.A. (1854). Revue de la famille des Urticacées. Ann. Sci. Natl. Bot. sér..

[bib97] Weddell H.A. (1856). Monographie de la famille des Urticées. Nouv. Archieves Mus. Hist. Natl..

[bib98] Weddell H.A., Candolle A.D. (1869). Prodromus Systematis Naturalis Regni Vegetabilis.

[bib99] Wells T., Maurin O., Dodsworth S. (2021). Combination of Sanger and target-enrichment markers supports revised generic delimitation in the problematic ‘*Urera* clade’ of the nettle family (Urticaceae). Mol. Phylogenet. Evol..

[bib100] Wilmot-Dear C.M. (1988). An account of the genus *Debregeasia* (Urticaceae-Boehmerieae). Kew Bull..

[bib101] Wilmot-Dear C.M. (2009). Urticaceae for the non-specialist: identification in the *Flora Malesiana* region, Indochina and Thailand. Blumea.

[bib102] Wilmot-Dear C.M., Friis I. (1996). The new world species of *Boehmeria* and *Pouzolzia* (Urticaceae, tribus Boehmerieae). A taxonomic revision. Oper. Bot..

[bib103] Wilmot-Dear C.M., Friis I. (1998). *Cypholophus decipiens* (Urticaceae): taxonomy and range of a species often misplaced in *Boehmeria*. Kew Bull..

[bib104] Wilmot-Dear C.M., Friis I. (2004). The old world species of *Pouzolzia* (Urticaceae, tribus Boehmerieae). A taxonomic revision. Nord. J. Bot..

[bib105] Wilmot-Dear C.M., Friis I. (2010). *Cypholophus anisoneurus* comb. nov. – an endemic species of Urticaceae from Vanuatu and Solomon Islands hitherto misplaced in *Boehmeria*. Nord. J. Bot..

[bib106] Wilmot-Dear C.M., Friis I., Thomas Z. (2010). New species in old world *Boehmeria* (Urticaceae). Edinb. J. Bot..

[bib107] Wilmot-Dear C.M., Friis I. (2012). *Debregeasia australis* sp. nov. (Urticaceae), with a new synopsis of and a new key to the genus. Edinb. J. Bot..

[bib108] Wilmot-Dear C.M., Friis I. (2013). The Old World species of *Boehmeria* (Urticaceae, tribus Boehmerieae). A taxonomic revision. Blumea.

[bib109] Wu Z.Y., Chapman M.A., Liu J. (2024). Genomic variation, environmental adaptation, and feralization in ramie, an ancient fiber crop. Plant Commun..

[bib110] Wu Z.Y., Liu J., Provan J. (2018). Testing Darwin's transoceanic dispersal hypothesis for the inland nettle family (Urticaceae). Ecol. Lett..

[bib111] Wu Z.Y., Milne R.I., Chen C.J. (2015). Ancestral state reconstruction reveals rampant homoplasy of diagnostic morphological characters in Urticaceae, conflicting with current classification schemes. PLoS One.

[bib112] Wu Z.Y., Milne R.I., Liu J. (2022). Phylogenomics and evolutionary history of *Oreocnide* (Urticaceae) shed light on recent geological and climatic events in SE Asia. Mol. Phylogenet. Evol..

[bib113] Wu Z.Y., Monro A.K., Milne R.I. (2013). Molecular phylogeny of the nettle family (Urticaceae) inferred from multiple loci of three genomes and extensive generic sampling. Mol. Phylogenet. Evol..

[bib114] Yang Y.Y., Qu X.J., Zhang R. (2021). Plastid phylogenomic analyses of Fagales reveal signatures of conflict and ancient chloroplast capture. Mol. Phylogenet. Evol..

[bib115] Yao G., Zhang Y.Q., Barrett C. (2023). A plastid phylogenomic framework for the palm family (Arecaceae). BMC Biology.

[bib116] Zhang C., Rabiee M., Sayyari E. (2018). ASTRAL-III: polynomial time species tree reconstruction from partially resolved gene trees. BMC Bioinformatics.

[bib117] Zhang C., Sayyari E., Mirarab S., Meidanis J., Nakhleh L. (2017). Comparative Genomics.

[bib118] Zhang R., Wang Y.H., Jin J.J. (2020). Exploration of plastid phylogenomic conflict yields new insights into the deep relationships of Leguminosae. Syst. Biol..

[bib119] Zhang S.D., Jin J.J., Chen S.Y. (2017). Diversification of Rosaceae since the Late Cretaceous based on plastid phylogenomics. New Phytol..

[bib120] Zhang S.D., Soltis D.E., Yang Y. (2011). Multi-gene analysis provides a well-supported phylogeny of Rosales. Mol. Phylogenet. Evol..

[bib121] Zhao F., Chen Y.P., Salmaki Y. (2021). An updated tribal classification of Lamiaceae based on plastome phylogenomics. BMC Biology.

[bib122] Zhao Y., Milne R.I., Li Z.P. (2024). *Boehmeria nivea* var. *strigosa* (Urticaceae), a new variety from Southwest China. Guihaia.

[bib123] Zhao Y., Milne R.I., Liu J. (2025). An integrated study of ramie (*Boehmeria nivea*), and its wild, cultivated, and feral forms. Ecol. Evol..

