# Supplementary material for: A robust phylogenomic framework supports a revised intrafamilial classification of Urticaceae
Source: Plant Divers. 2025 Dec 17;48(2):289–306. doi: 10.1016/j.pld.2025.12.003 (PMC13071455; doi:10.1016/j.pld.2025.12.003)

Percentage length recovery for each gene, relative to mean of targetfile references

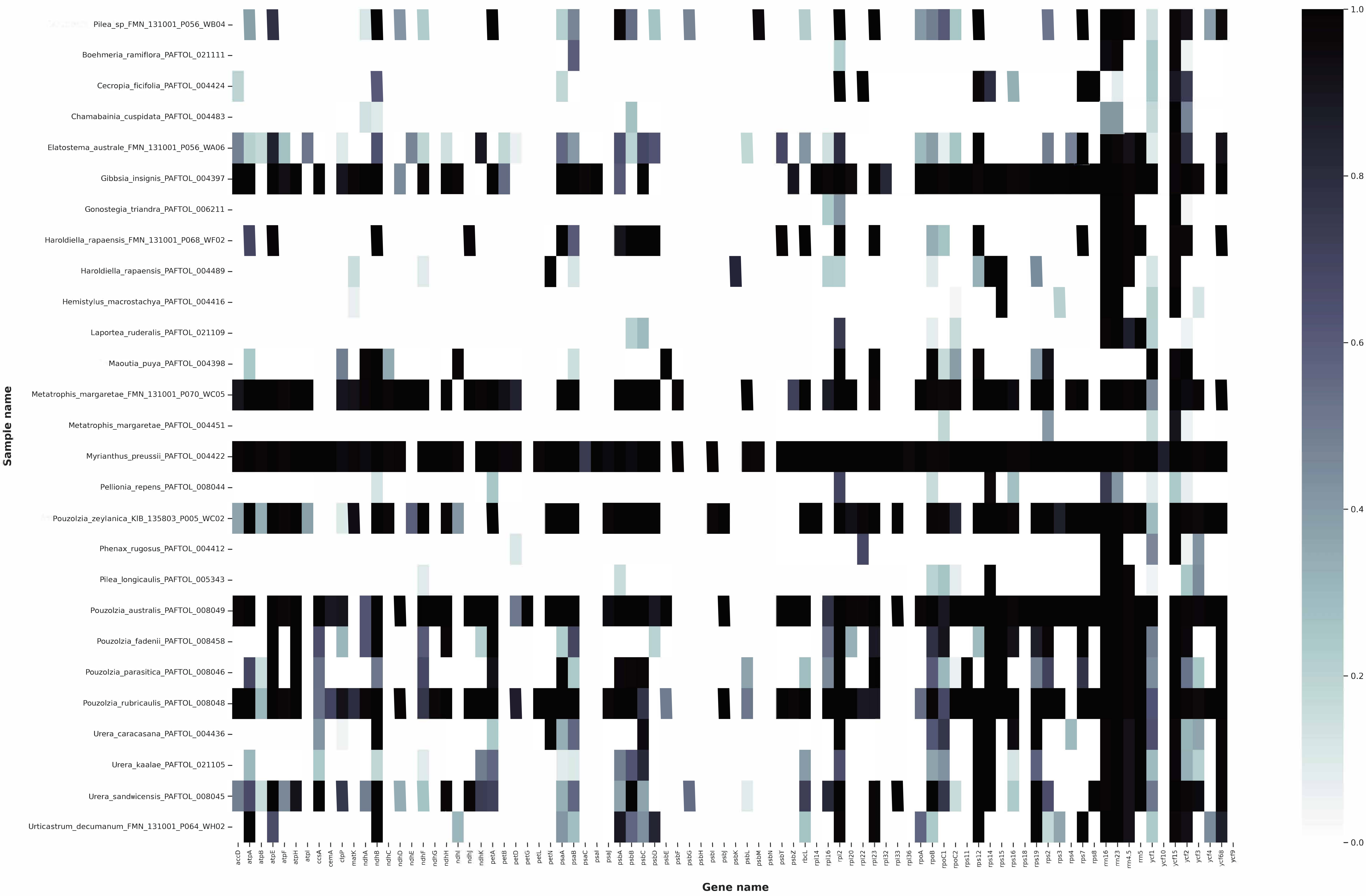

Supplement: Multimedia component 1 [file mmc1.pdf]
