# Supplementary figures and images for: A robust phylogenomic framework supports a revised intrafamilial classification of Urticaceae
Source: Plant Divers. 2025 Dec 17;48(2):289–306. doi: 10.1016/j.pld.2025.12.003 (PMC13071455; doi:10.1016/j.pld.2025.12.003)

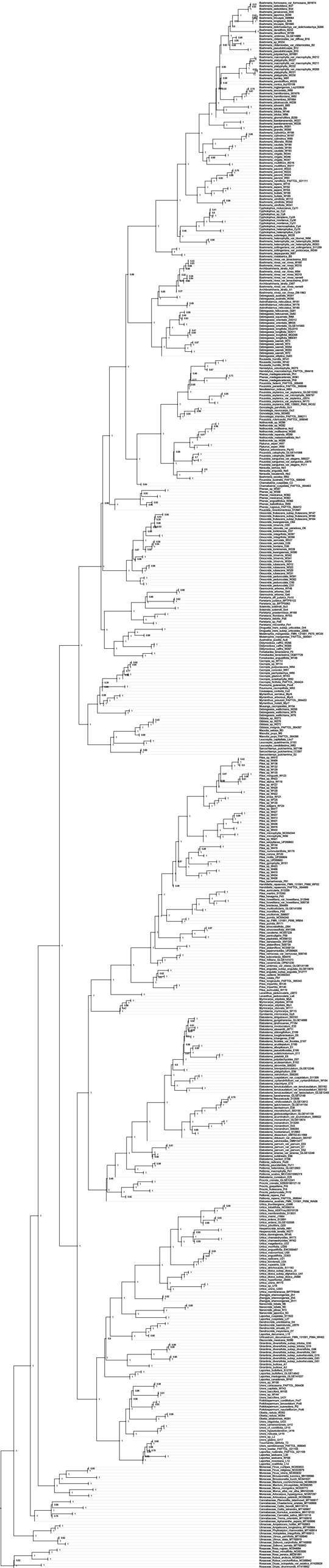

Supplement: Multimedia component 2 [file mmc2.pdf]

**RAxML tree**

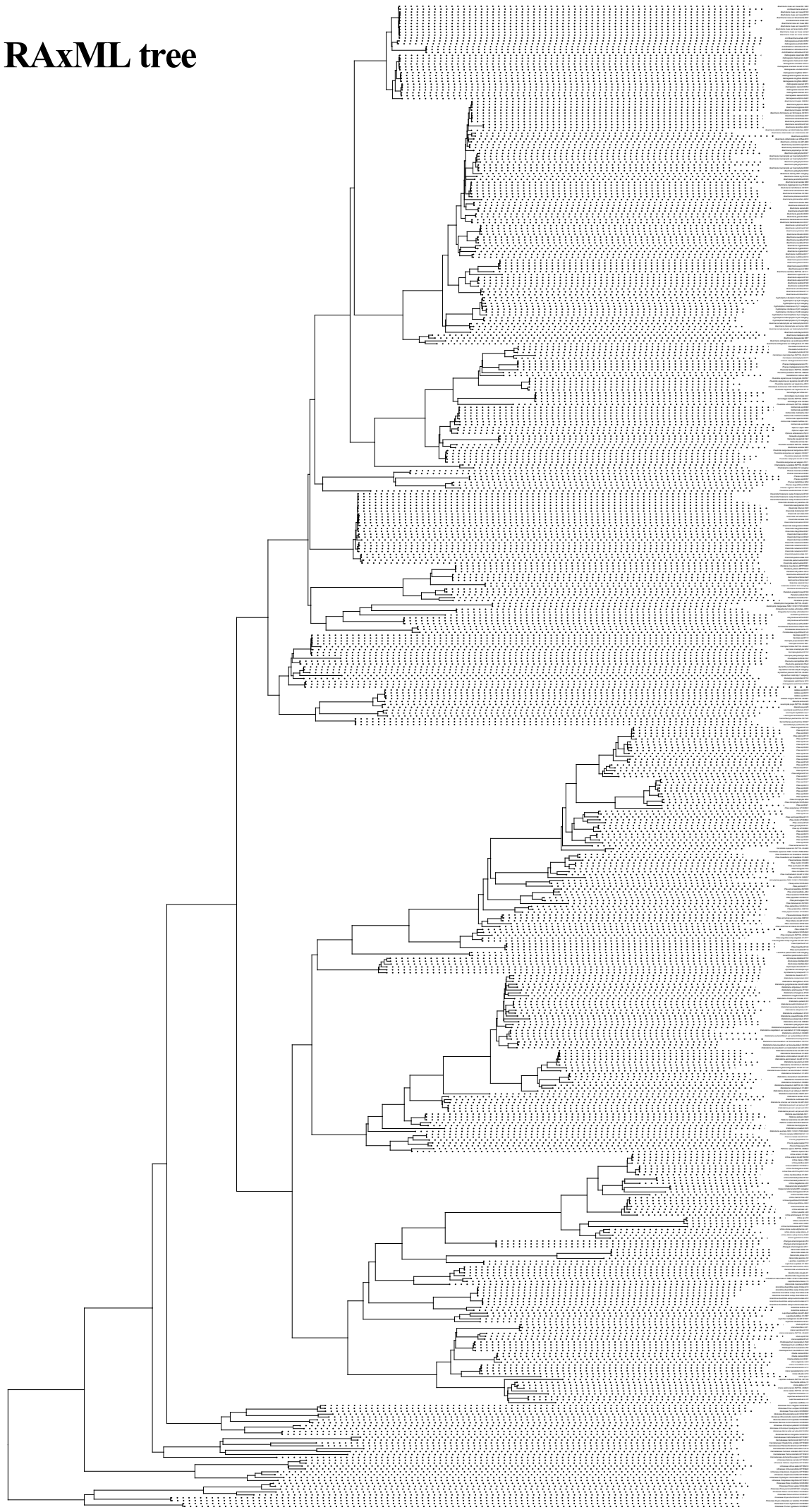

**ASTRAL tree**

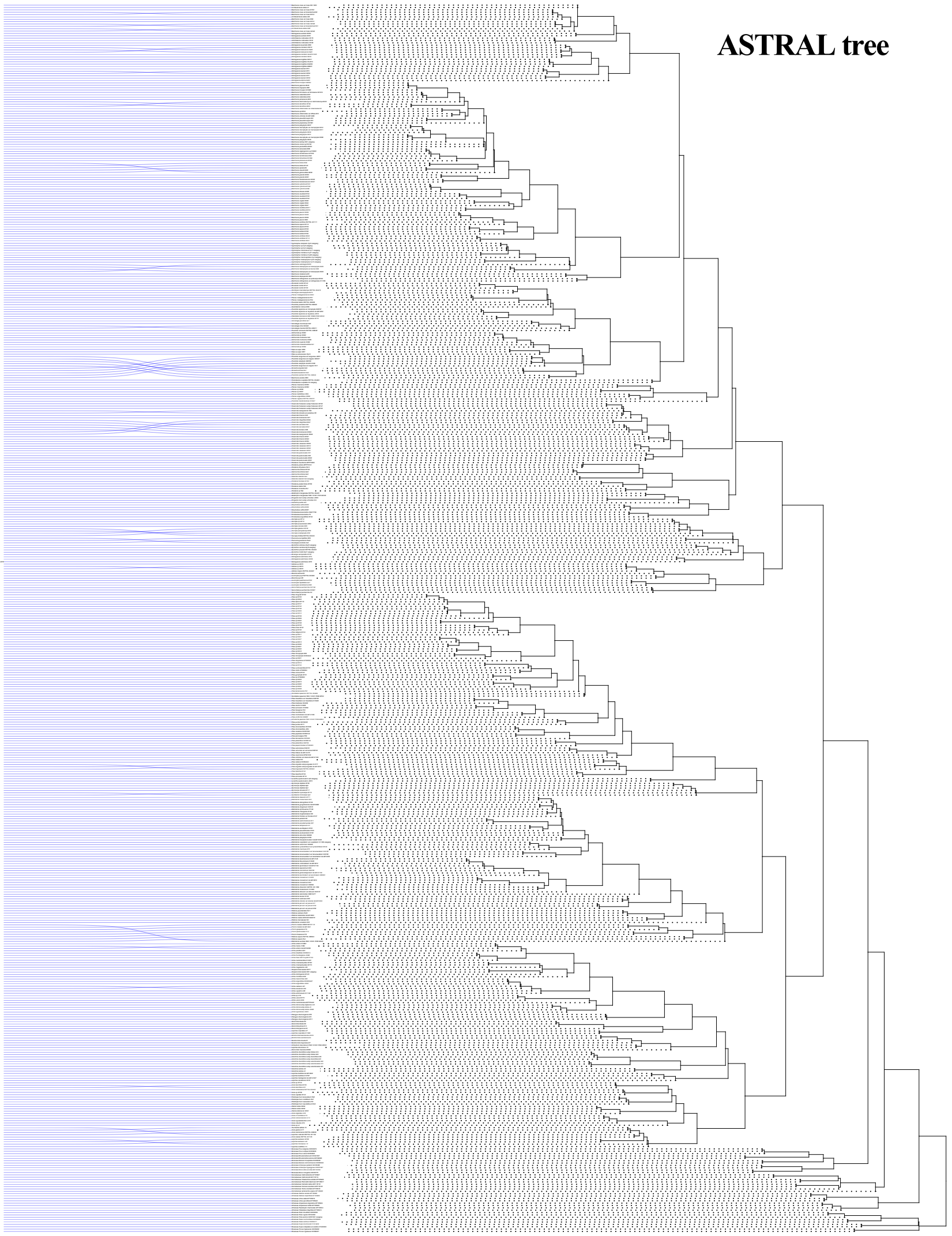

Supplement: Multimedia component 6 [file mmc6.pdf]

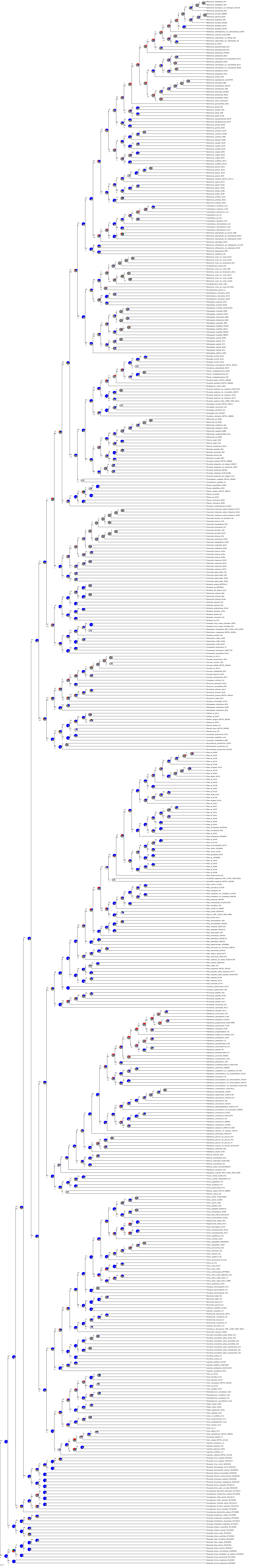

Supplement: Multimedia component 7 [file mmc7.pdf]

(A) Wu et al. 2018

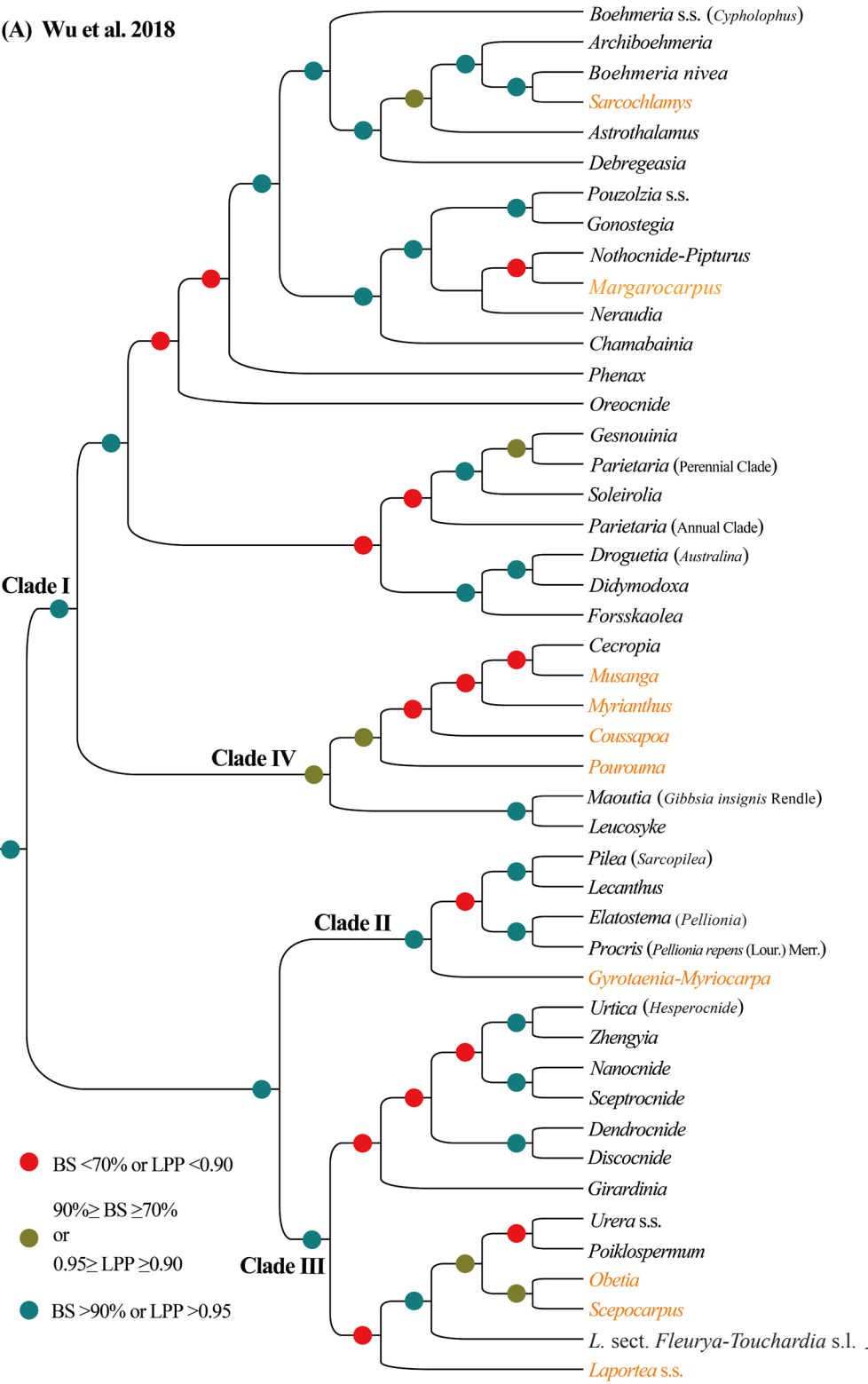

(B) This study

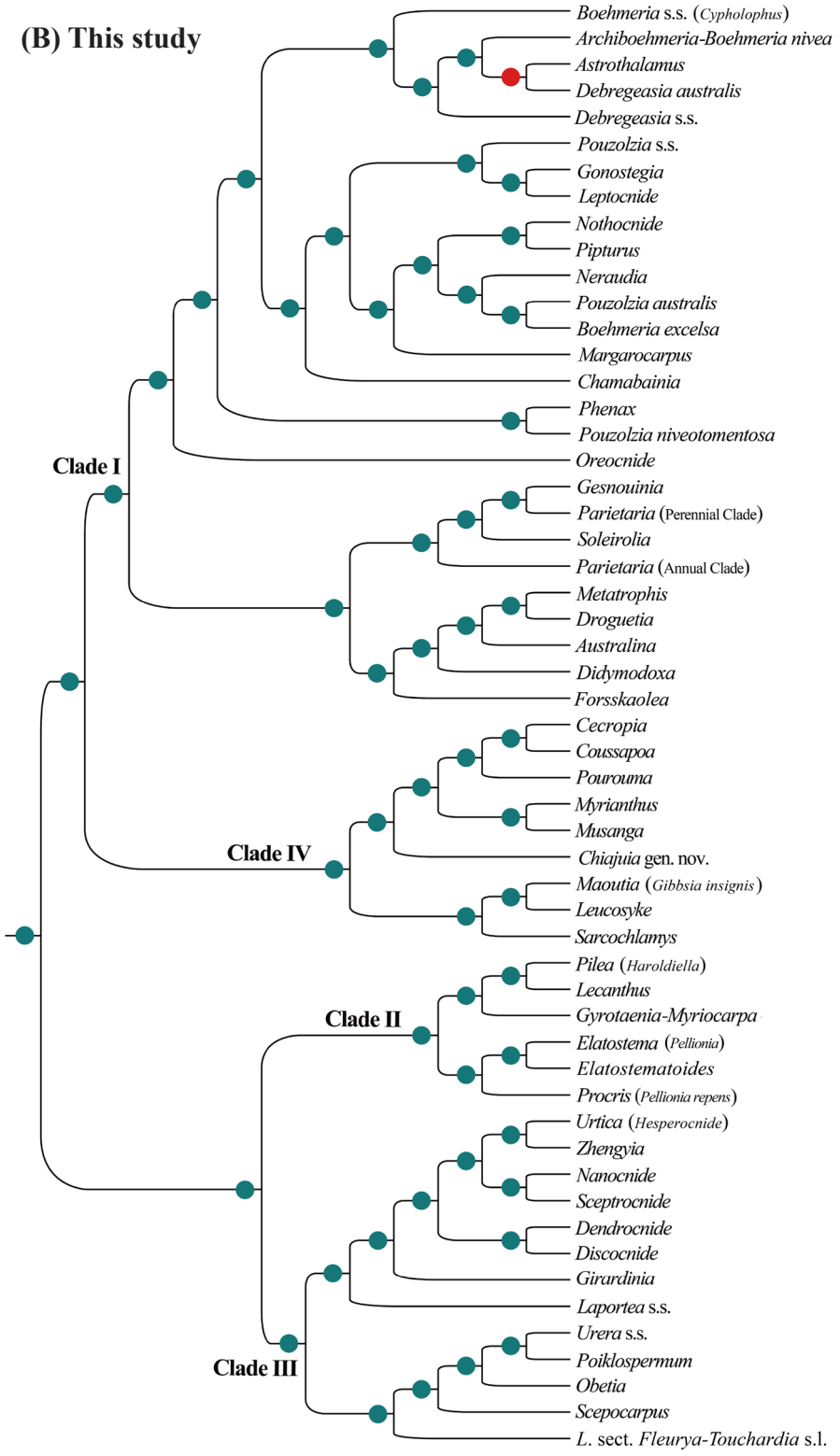

Supplement: Multimedia component 10 [file mmc10.pdf]

(A) Wu et al. 2018

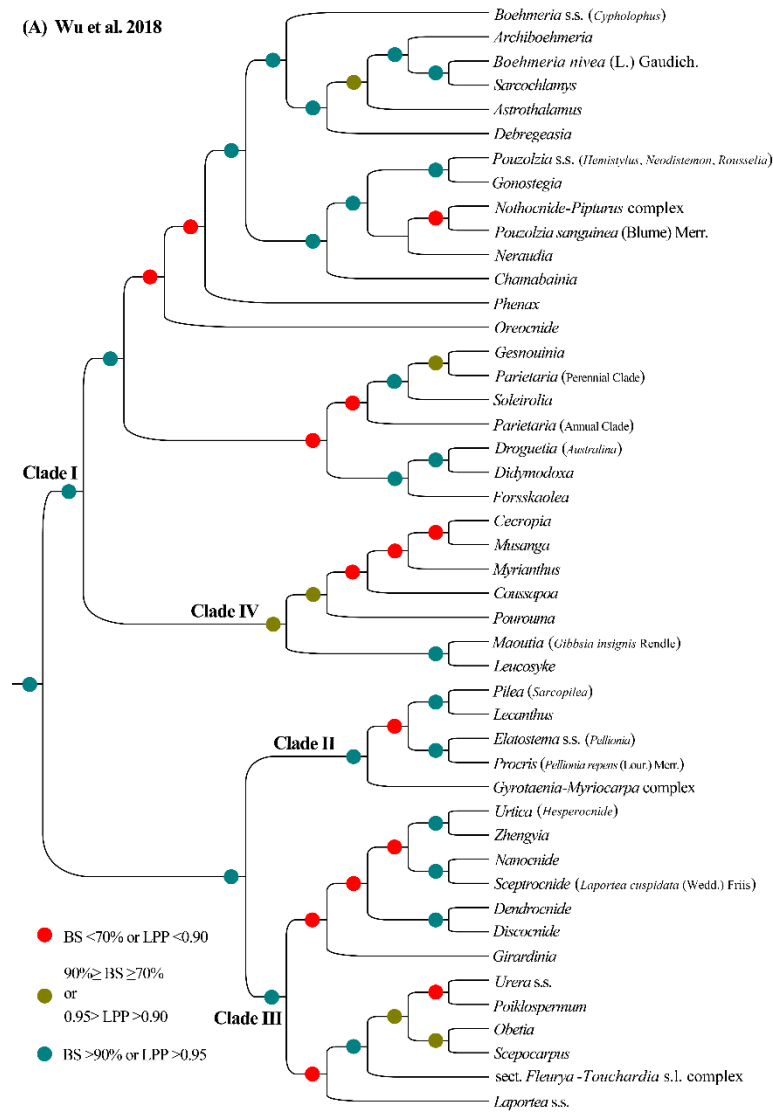

(B) This study

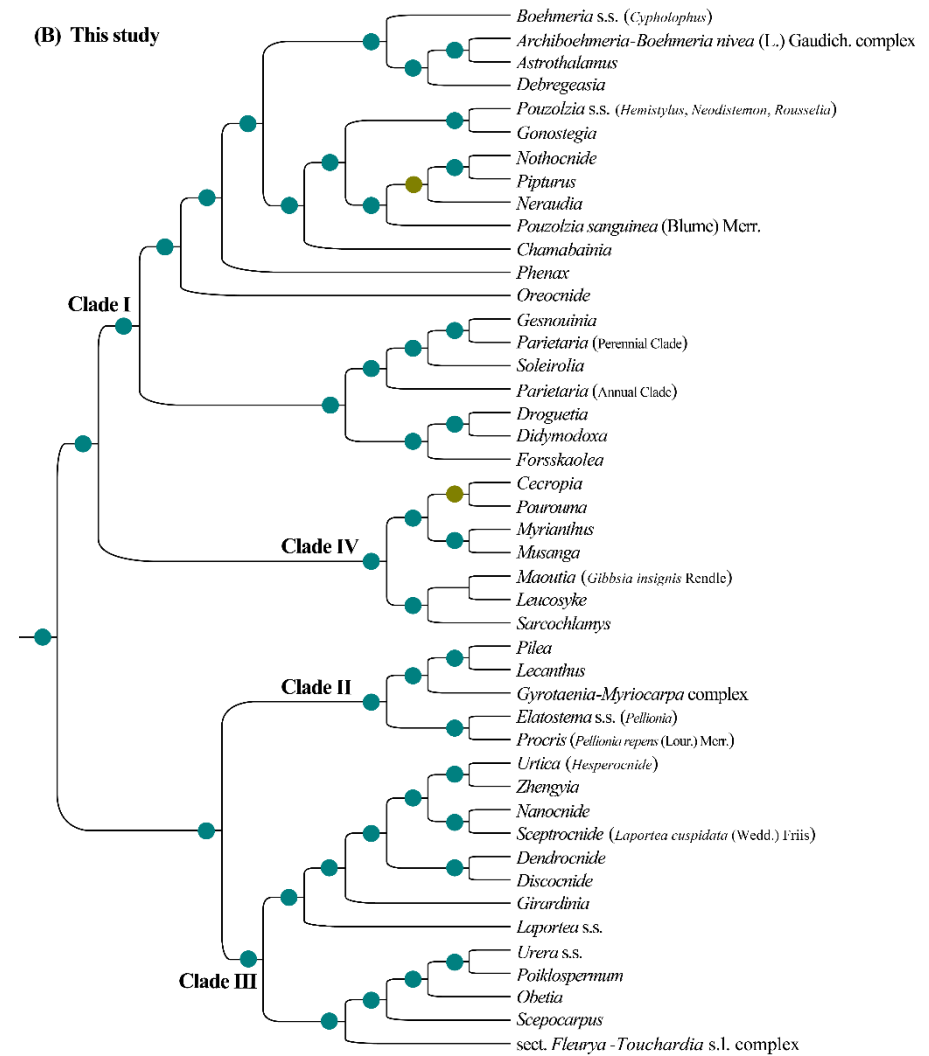

Supplement: Multimedia component 11 [file mmc11.pdf]
