# Supplementary material for: A robust phylogenomic framework supports a revised intrafamilial classification of Urticaceae
Source: Plant Divers. 2025 Dec 17;48(2):289–306. doi: 10.1016/j.pld.2025.12.003 (PMC13071455; doi:10.1016/j.pld.2025.12.003)
Supplement: Multimedia component 9 [file mmc9.pdf]

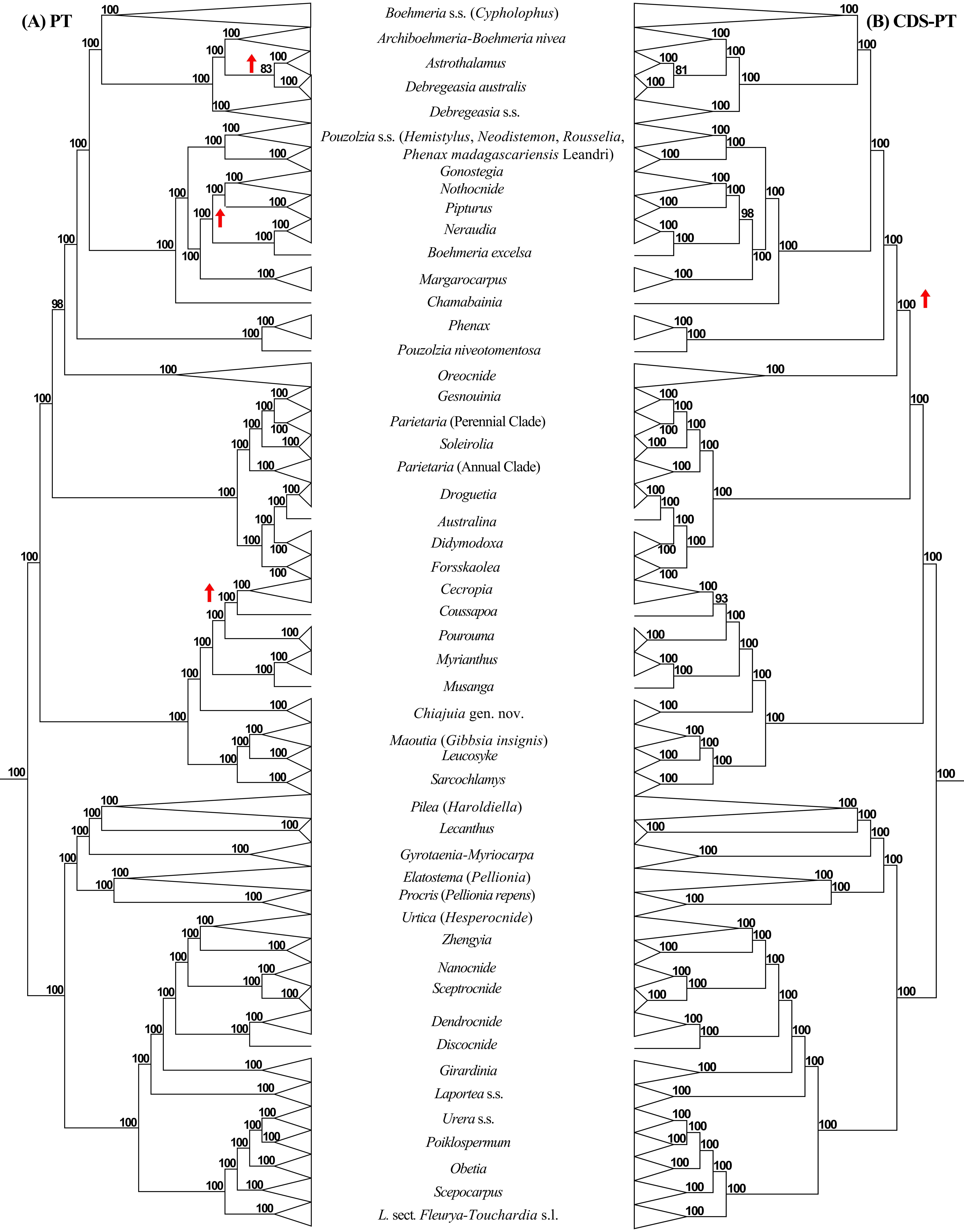

(A) PT
(B) CDS-PT
Boehmeria s.s. (Cypholophus)
Archiboehmeria-Boehmeria nivea
Astrothalamus
Debregeasia australis
Debregeasia s.s.
Pouzolzia s.s. (Hemistylus, Neodistemon, Rousselia,
Phenax madagascariensis Leandri)
Gonostegia
Nothocnide
Pipturus
Neraudia
Boehmeria excelsa
Margarocarpus
Chamabainia
Phenax
Pouzolzia niveotomentosa
Oreocnide
Gesnouinia
Parietaria (Perennial Clade)
Soleirolia
Parietaria (Annual Clade)
Droguetia
Australina
Didymodoxa
Forsskaolea
Cecropia
Coussapoa
Pourouma
Myrianthus
Musanga
Chiajuia gen. nov.
Maoutia (Gibbsia insignis)
Leucosyke
Sarcochlamys
Pilea (Haroldiella)
Lecanthus
Gyrotaenia-Myriocarpa
Elatostema (Pellionia)
Procris (Pellionia repens)
Urtica (Hesperocnide)
Zhengyia
Nanocnide
Sceptrocnide
Dendrocnide
Discocnide
Girardinia
Laportea s.s.
Urera s.s.
Poikilospermum
Obetia
Scepocarpus
L. sect. Fleurya-Touchardia s.l.
