# Supplementary material for: A robust phylogenomic framework supports a revised intrafamilial classification of Urticaceae
Source: Plant Divers. 2025 Dec 17;48(2):289–306. doi: 10.1016/j.pld.2025.12.003 (PMC13071455; doi:10.1016/j.pld.2025.12.003)
Supplement: Multimedia component 12 [file mmc12.docx]

| **Previous_studies** | **Major_division_within_Urticaceae** | | **No_of_genus** | **Names_of_genus** |
| --- | --- | --- | --- | --- |
| Jussieu 1789 | Urticae II | – | 13 | *Artocarpus, Boehmeria, Cannabis, Cecropia, Elatostema, Forskalea, Humulus, Morus, Parietaria, Procris, Pteranthus, Theligonum, Urtica* |
| Gaudichaud 1830 | – | Trib. 1. Boehmerieae | 3 | *Boehmeria, Neraudia, Procris* |
|  | – | Trib. 2. Cecropieae | 2 | *Cecropia, Coussapoa* |
|  | – | Trib. 3. Elatostemeae | 5 | *Dubrueilia, Elatostema, Langeveldia, Pellionia, Sciophila* |
|  | – | Trib. 4. Forskalieae | 3 | *Australina, Droguetia, Forskalea* |
|  | – | Trib. 5. Parietarieae | 7 | *Freirea, Gesnouinia, Parietaria, Pouzolzia, Rousselia, Soleirolia, Thaumuria* |
|  |  | Trib. 6. Urereae | 5 | *Fleurya, Girardinia, Laportea, Urera, Urtica* |
| Weddell 1854, 1856 & 1869 | – | Trib. 1. Boehmerieae | 16 | *Boehmeria, Chamabainia, Cypholophus, Laurea, Leucosyke, Poikilospermum, Pouzolzia, Maoutia, Memorialis, Myriocarpa, Neraudia, Phenax, Pipturus, Sarcochlamys, Touchardia, Villebrunea* |
|  | – | Trib. 2. Forskahleae | 5 | *Australina, Didymodoxa, Distemon, Droguetia, Forskohlea* |
|  | – | Trib. 3. Parietarieae | 5 | *Gesnouinia, Helxine, Hemistylis, Parietaria, Rousselia* |
|  | – | Trib. 4. Procrideae | 6 | *Achudemia, Elatostema, Lecanthus, Pellionia, Pilea, Procris* |
|  | – | Trib. 5. Urereae | 10 | *Fleurya, Girardinia, Gyrotaenia, Hesperocnide, Laportea, Nanocnide, Obetia, Scepocarpus, Urera, Urtica,* |
| Friis 1989 &1993 | – | Trib. 1. Boehmerieae | 19 | *Astrothalamus, Boehmeria, Chamabainia, Cypholophus, Debregeasia, Gibbsia, Gonostegia, Leucosyke, Maoutia, Myriocarpa, Neodistemon, Neraudia, Nothocnide, Oreocnide, Phenax, Pipturus, Pouzolzia, Sarcochlamys, Touchardia* |
|  | – | Trib. 2. Forsskaoleae | 4 | *Australina, Didymodoxa, Droguetia, Forsskaolea* |
|  | – | Trib. 3. Lecantheae | 7 | *Elatostema, Lecanthus, Meniscogyne, Petelotiella, Pilea, Procris, Sarcopilea* |
|  | – | Trib. 4. Parietarieae | 5 | *Gesnouinia, Hemistylus, Parietaria, Rousselia, Soleirolia* |
|  | – | Trib. 5. Urticeae | 10 | *Dendrocnide, Discocnide, Girardinia, Gyrotaenia, Hesperocnide, Laportea, Nanocnide, Obetia, Urera, Urtica* |
| Kravtsova 2007 & 2009 | Subfam. 1. Urticoideae | Trib. 1. Urticeae | 10 | *Dendrocnide, Discocnide, Girardinia, Gyrotaenia, Hesperocnide, Laportea, Nanocnide, Obetia, Urera, Urtica* |
|  | Subfam. 2. Lecanthoideae | Trib. 2. Lecantheae | 7 | *Achudemia, Elatostema, Lecanthus, Meniscogyne, Pellionia, Pilea, Procris* |
|  |  | Trib. 3. Touchardieae | 1 | *Touchardia* |
|  | Subfam. 3. Boehmerioideae | Trib. 4. Boehmerieae | 20 | *Archiboehmeria, Astrothalamus, Boehmeria, Chamabainia, Cypholophus, Debregeasia, Gibbsia, Hemistylus, Leucosyke, Maoutia, Myriocarpa, Neodistemon, Neraudia, Nothocnide, Oreocnide, Phenax, Pipturus, Pouzolzia, Rousselia, Sarcochlamys* |
|  |  | Trib. 5. Forsskaoleae | 4 | *Australina, Didymodoxa, Droguetia, Forsskaolea* |
|  |  | Trib. 6. Parietarieae | 3 | *Gesnouinia, Parietaria, Soleirolia* |
| Hadiah 2008 | Boehmeriea–Cecropieae–Forsskaoleeae–Parietarieae lineage | – | 16 | *Boehmeria, Cecropia, Coussapoa, Cypholophus, Debregeasia, Didymodoxa, Droguetia, Forsskaolea, Gesnouinia, Gonostegia, Leucosyke, Maoutia, Nothocnide, Oreocnide, Parietaria, Pouzolzia,* |
|  | Urticeae lineage | – | 6 | *Dendrocnide, Discocnide, Laportea, Poikilospermum, Urera, Urtica* |
|  | Elatostemeae lineage | – | 6 | *Elatostema, Lecanthus, Myriocarpa, Pellionia, Pilea, Procris* |
| Wu et al. 2013 & 2018 | Clade I | – | 24 | *Archiboehmeria, Astrothalamus, Australina, Boehmeria, Chamabainia, Cypholophus, Debregeasia, Didymodoxa, Droguetia, Forsskaolea, Gesnouinia, Gonostegia, Hemistylus, Neodistemon, Neraudia, Nothocnide, Oreocnide, Parietaria, Phenax, Pipturus, Pouzolzia, Rousselia, Sarcochlamys, Soleirolia* |
|  | Clade II | – | 8 | *Elatostema, Gyrotaenia, Lecanthus, Myriocarpa, Pilea, Pellionia, Procris, Sarcopilea* |
|  | Clade III | – | 12 | *Dendrocnide, Discocnide, Girardinia, Hesperocnide, Laportea, Nanocnide, Obetia, Poikilospermum, Touchardia, Urera, Urtica, Zhengyia* |
|  | Clade IV | – | 8 | *Cecropia, Coussapoa, Gibbsia, Leucosyke, Maoutia, Musanga, Myrianthus, Pourouma* |
