# Supplementary material for: A robust phylogenomic framework supports a revised intrafamilial classification of Urticaceae
Source: Plant Divers. 2025 Dec 17;48(2):289–306. doi: 10.1016/j.pld.2025.12.003 (PMC13071455; doi:10.1016/j.pld.2025.12.003)
Supplement: Multimedia component 4 [file mmc4.pdf]

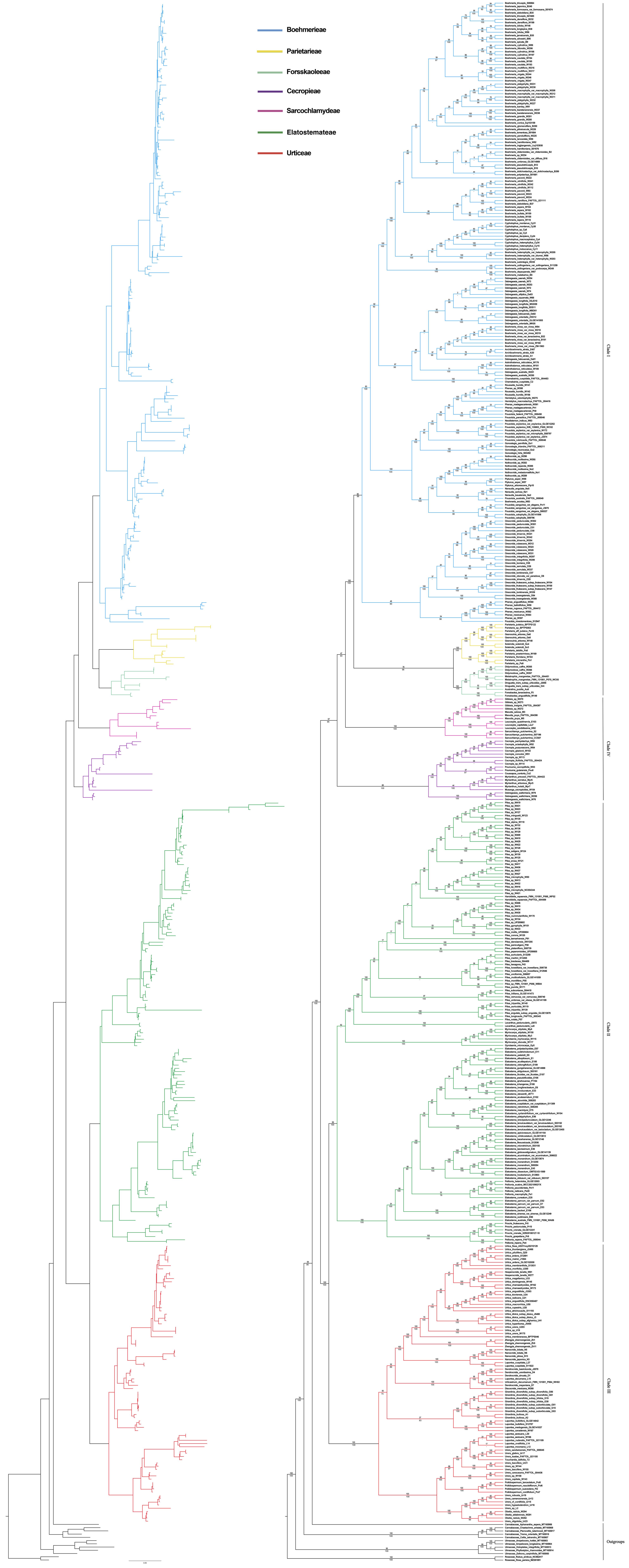

Boehmerieae
Parietarieae
Forsskaoleeae
Cecropieae
Sarcochlamydeae
Elatostemateae
Urticeae
Clade I
Clade IV
Clade II
Clade III
Outgroups
